# Inventing the Grand Banks: A deep chart: Humanities GIS, Cartesian, and literary perceptions of the north‐west Atlantic fishery ca 1500–1800

**DOI:** 10.1002/geo2.85

**Published:** 2020-03-30

**Authors:** Charles Travis, Francis Ludlow, Al Matthews, Kevin Lougheed, Kieran Rankin, Bernard Allaire, Robert Legg, Patrick Hayes, Richard Breen, John Nicholls, Lydia Towns, Poul Holm

**Affiliations:** ^1^ Department of History University of Texas Arlington Texas; ^2^ Department of History Trinity College Dublin Dublin Ireland; ^3^ Trinity Centre for Environmental Humanities University of Dublin Trinity College Dublin Ireland

**Keywords:** Benjamin Franklin’s *Gulp Stream Chart*, Cabot Voyage, Deep Chart, Fish Revolution 1500-1800, Grand Banks, Humanities GIS, Shakespeare’s *The Tempest*

## Abstract

As a feature of the Fish Revolution (1400–1700), the early modern “invention” of the Grand Banks in literary and cartographical documents facilitated a massive and unprecedented extraction of cod from the waters of the north Atlantic and created the Cod/Sack trade Triangle. This overlapped with the southern Atlantic Slave, Sugar, and Tobacco Triangle to capitalise modern European and North American societies. In 1719, Pierre de Charlevoix claimed that the Grand Banks was “properly a mountain, hid under water,” and noted its cod population “seems to equal that of the grains of sand which cover this bank.” However, two centuries later in 1992, in the face of the collapse of the fishery, and fearing its extinction, a moratorium was placed on five centuries of harvesting Grand Banks cod. The invention and mining of its waters serves as a bellwether for the massive resource extractions of modernity that drive the current leviathan and “wicked problem” of global warming. The digital environmental humanities narrative of this study is parsed together from 83 pieces of Grand Banks charting from 1504 to 1833, which are juxtaposed through Humanities GIS applications with English and French cod‐catch records kept between 1675 and 1831, letters regarding Cabot's 1497 voyage, Shakespeare's *The Tempest* (1611) and scientific essays by De Brahms (1772) and Franklin (1786).

## Graphical abstract

The early modern “invention” of the Grand Banks in literary and cartographical documents contributed to facilitating a massive and unprecedent extraction of cod from the waters of the north Atlantic and created the Cod/Sack trade Triangle. This feature of the Fish Revolution (1400–1700) over lapped with the southern Atlantic Slave, Sugar and Tobacco Triangle in its contribution to the capitalization of modern European and North America societies. In 1719, Pierre de Charlevoix claimed that the Grand Banks was “properly a mountain, hid under water,” and noted its cod population “seems to equal that of the grains of sand which cover this bank.” However, two centuries later in 1992, in the face of the collapse of the fishery, and fearing its extinction, a moratorium was placed on five centuries of harvesting cod from north‐west Atlantic waters. This study examines 83 Grand Banks charts drafted between 1504 to 1833, and digitally contextualizes the morphology of their fishery symbolism over three hundred years with English and French cod‐catch records (1675–1831), epistolary accounts of John Cabot’s accidental discovery of a massive shoal of Gadus morhua (cod) during his 1497 voyage to a “newfoundland”; the influence of the emerging Grand Banks fishery on William Shakespeare’s The Tempest (1611) and scientific essays on the oceanic system of the Gulf Stream by William Gerard De Brahm (1772) and Benjamin Franklin (1786). This study illustrates through methods in digital hermeneutics and humanities GIS how an unprecedent extraction of cod from north‐west Atlantic waters influenced early modern cartographical, literary, and scientific perceptions, discourses and practices, situating the Grand Banks as an invention of the Fish Revolution resulting from the confluence of European and North American maritime culture that shaped the course of modern transatlantic political‐economic development and relations.

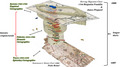

Where is your tribal memory? Sirs,

In that gray vault. The sea. The sea

Has locked them up. The sea is History.

Derek Walcott, *The Sea is History* (2007)

## 1 INTRODUCTION

The early modern “invention” of the Grand Banks, a feature of the Fish Revolution (ca 1500), facilitated massive and unprecedented extractions of *Gadus morhua* (cod) from the waters of the north Atlantic. The invention of this oceanic plantation, the result of a confluence between cartography, commerce, and cultural agency, contributed to capitalising modern European and North American societies, and created conflict between bourgeoning Western empires. Fernand Braudel noted that “a historical study centred on a stretch of water has all the charms but undoubtedly all the dangers of a new departure” (1973, p. 19). Embracing such a risk, this paper engages Humanities GIS (HumGIS) as a method to parse knowledge from sea and fishery charts, letters, pieces of drama, and scientific surveys in order to study the historical geographies of the early modern north Atlantic *économie‐monde.*

**Figure 1:**
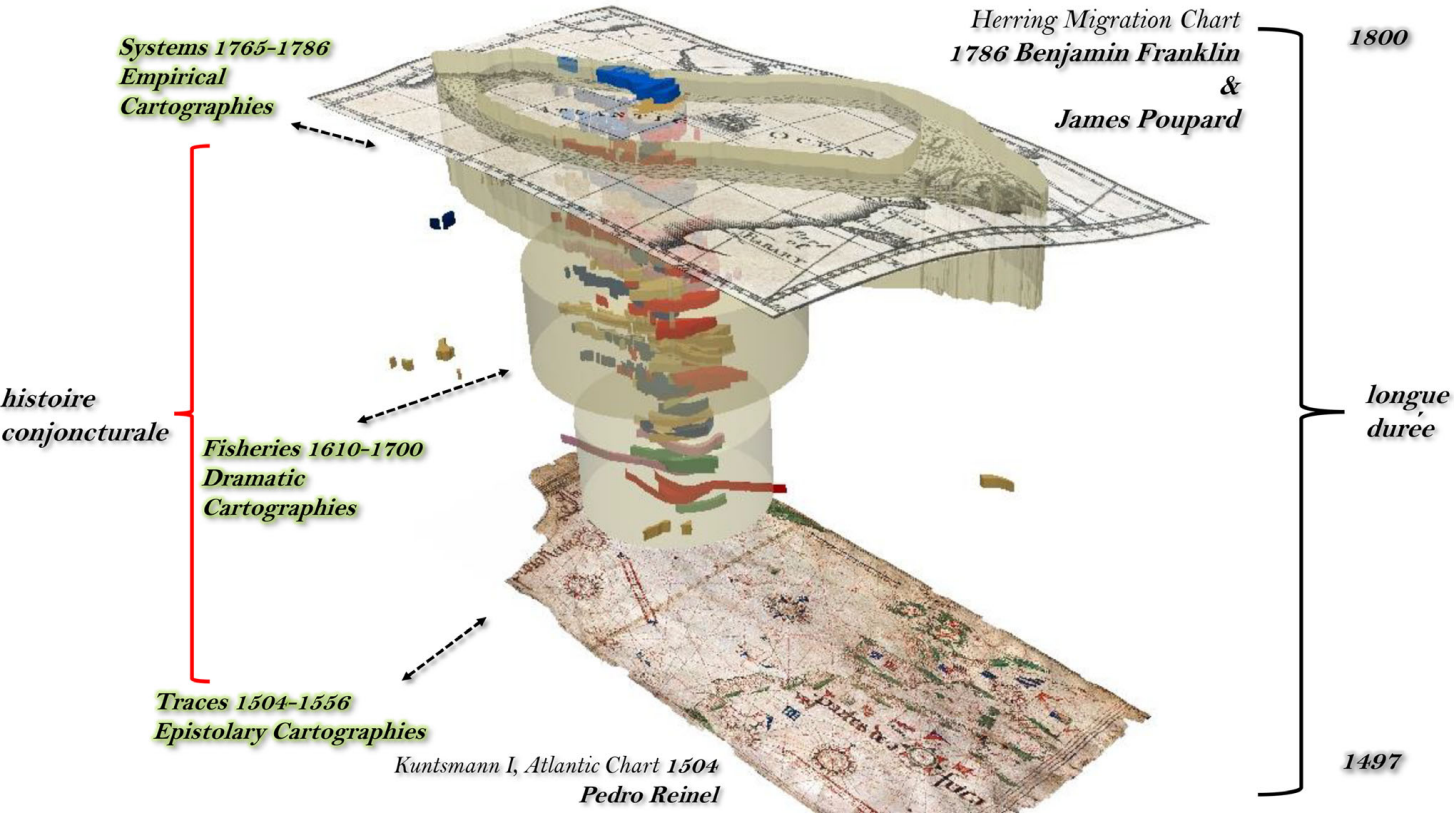
The Grand Banks Deep Chart: Le longue durée and its three histoire conjoncturelles: (a) traces, (b) fisheries, (c) systems (Charles Travis, Kevin Lougheed, Francis Ludlow).

Evolution in Grand Banks cartography over the *longue durée* is marked by three Braudelian *histoire conjoncturelle* (historical cycles). Charts of the north‐west Atlantic in the first cycle appear as portolan navigational line *tracings* (1504–1556). In the second cycle the modern extent of the Grand Banks *fisheries* emerge and, in the English case, can be contextualised by Shakespearean drama and the Thames School of Cartography (1600–1700). Third cycle charts begin to reflect scientific understandings of the *systems* of ocean currents, such as the Gulf Stream that drove the Atlantic trade triangles (1765–1786). Under the lens of a HumGIS, *distant readings* of cod catch/fishery symbolism cartography (1675–1831) correlations were parsed in juxtaposition with *close readings* of texts representing three historic‐cartographical cycles of the Fish Revolution: first, epistolary accounts of John Cabot's (aka Zuan Chabotto) 1497 north‐west Atlantic voyage; second, a geographical exegesis of Shakespeare's *The Tempest* (1611); and third, scientific essays on empirical surveys of the Gulf Stream by William Gerard De Brahm in *The Atlantic Pilot* (1772) and in Benjamin Franklin's *Sundry Maritime Observation* (1786)^1^ (see Figure 1).

Integrating oceanic charts and literature in a digital hermeneutic (click to access the Inventing the Grand Banks Deep Chart digital timeline map) this paper builds upon over a century's worth of geographical literature on the sea. Studies range from oceanic physiography and mobility, to medieval and modern trade; from political, historical, social, and cultural geographies of the Atlantic, Caribbean, Polar, Pacific, and Indian oceans, to ships, sailors, and shipwrecks (Andrews, 1955; Hasty & Peters, 2012; le Messurier, 1916; Mancke, 1999; Ogborn, 2005; Peters, 2010; Smith, 1968; Steinberg, 2009; Wharton, 1894). More recently, GIS, digital cartography, humanities, and qualitative data analysis studies have focused on visual, data led, and discursive remediations of river, estuary, harbour, and oceanic spaces, charts, and visual art; voyages of discovery, encounter, and colonial mercantile and port networks; sea‐fishing, parliamentary papers, ship's logs, and navigation routes; geographical phenomena of land and sea relations in law and literature, and “Blue Economy” geo‐imaginaries (Beard et al., 2014; Blundell & Zernecke, 2014; Buck, 2009; Manca & Waters, 2017; Manderson & van Rijswijk, 2015; Pearce & Hermann, 2010; Rivera Medina, 2017; Schmidt, 2012; Schwartz, 2015; Solana, 2014; Southall, 2013; Winder & Le Heron, 2017).

**Figure 2:**
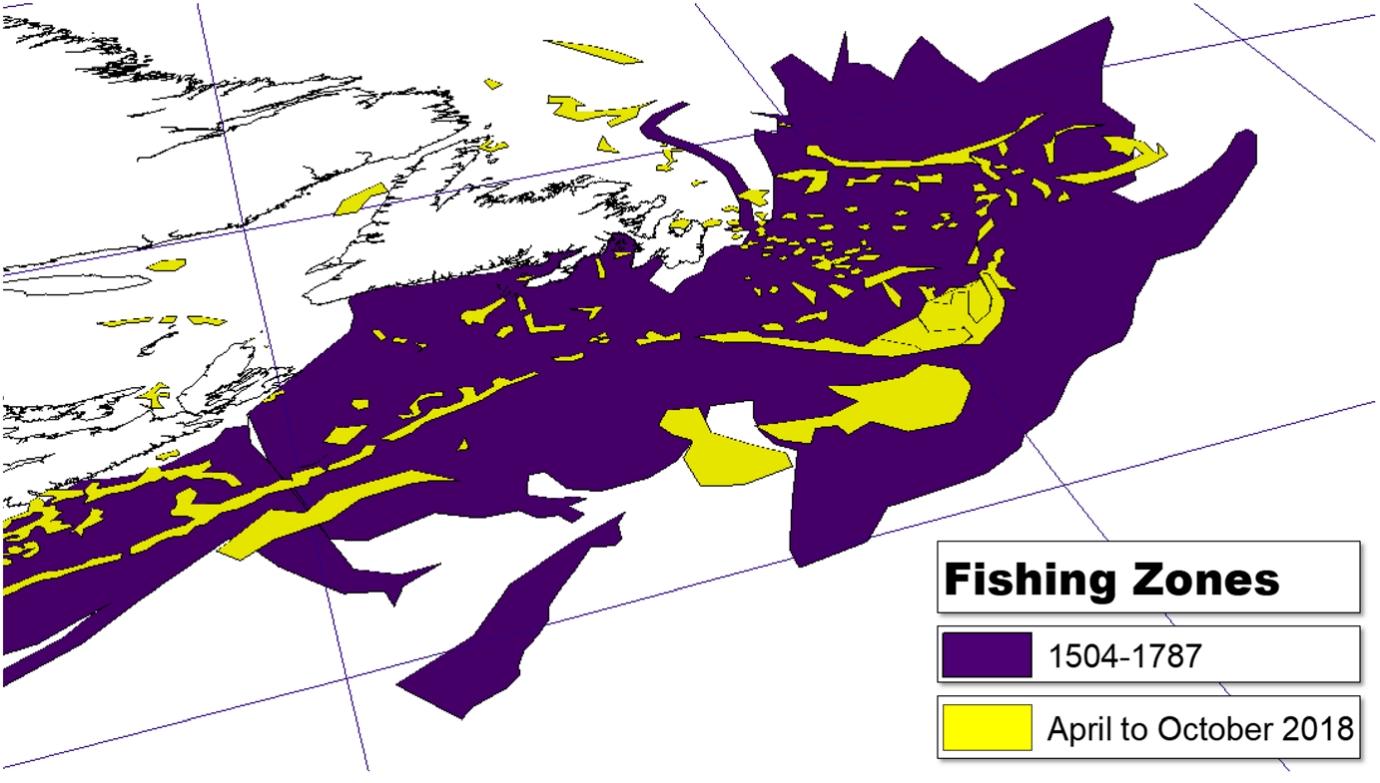
Fishing zones: 1504–1787 and April–October 2018 (Charles Travis, Kevin Lougheed, Francis Ludlow, Robert Legg).

In 1719, the Jesuit historian Pierre de Charlevoix contended that the cod fisheries of the Grand Banks were the “true mines, which are the more valuable, and require much less expence [sic] than those of Peru and Mexico” (Roberts, 2007, p. xxvii). However, the “mining” of its waters serves as a bellwether for the massive resource extractions of modernity that drive the current “wicked problem” of global warming. Intimations of a collapse of the Grand Banks fisheries were heard “as early as 1703 [when] an Englishman lamented from coastal Newfoundland that ‘the fish grows less, the old store being consumed by our continual fishing’” (Bolster, 2006, p. 568; see alo Figure 2).

Just over two centuries later, in July 1992, the Canadian Government, fearing the extinction of the fishery, placed a moratorium on five centuries of harvesting Grand Banks cod. This resulted in the single largest mass layoff in Canadian history, affecting fishing crews, workers in processing plants, global fishmongers, and shipyards (Organization for Economic Co‐operation & Development, 2011). The difference illustrated in Figure 2 of the expanse of early modern Grand Bank fishing zones charted between 1504 and 1787, with fishing zones operating in 2018 can be attributed to many factors: the collapse of the fishery and moratorium on cod, as well as technological innovations in oceanic cartography. However, commensurate species collapses occurred after the European settlement of North America. Passenger pigeon (*Ectopistes migratorius*) flock sizes spanning the distance between New York City and Philadelphia began to be harvested from 1800 in numbers estimated at four million pigeons a year. The species became extinct in 1914. American bison (*bison*) herds ranging from the tide‐water Atlantic to the northern Great Plains were estimated to number 30 million in the 16th century; by the 1870s the species was decimated to 1091 (Foster, 2014; Hornaday, 1899; Stanton, 2014).

### 1.1 Deep charting

To explore the stories behind the collapse of the north‐west Atlantic cod fishery, a deep chart model of the Grand Banks plotting the geographical and historical antecedents of this human‐environmental catastrophe was created. Grounded (like its terrestrial cousin “deep mapping”) in “eighteenth‐century antiquarian approaches to geography, history, people, culture, and place” (Harris, 2015, p. 42), deep charting explores the “more quantifiable signatures of a specific region,” whilst “counter‐mapping or reclaiming the map itself” (Hayman et al., 2017, p. 236). The method has also been engaged “to construct new separate/connected/intersecting spatial narratives” by expanding or re‐interpreting “existing ones already embedded within the deep map framework” (Oxx et al., 2013, pp. 206–207). Literary scholar Franco Moretti's *distant reading* method, focusing on “units that are much smaller or much larger than the text: devices, themes, tropes ‐or genres and systems” (2013, p. 48‐49), was juxtaposed in HumGIS with *close readings* of charts and contextual literature. Such readings engaged the “textuality of history, and historicity of texts,” recognising that charts and literature “cannot be abstracted from the historical context” in which they were “produced and consumed” (Wiener, 1988, p. 621).

HumGIS mapped the specific cycles of history in which charts, letters, pieces of drama, and scientific surveys were located over the Braudelian *longue durée* (the glacial passage of geographical time, in which humans interact with environments for survival). The readings focused acutely on the personalities and unique sea‐changing events (*histoire événementielle*) occurring with the specific historical cycles (*histoire conjoncturelle*). The application of *distant* and *close* readings within such Braudelian temporal scales complements recent narrative turns in geography and the study of chronology and dating to provide thick, rather than thin, portraits of time and place (Daniels & Bartlein, 2017).

### 1.2 The fish revolution

Arguably, the liminal “oceanic space” of the Grand Banks had as much a long‐term impact as the iconic Virginia and Roanoke colonial settlements in capitalising the political economies of early modern North America and Western Europe. As a trans‐national, and seemingly endless supply of cod, its fishery provided unprecedented commodity and capital flows for English and European fish brokers and markets. Cabot's 1497 voyage, sponsored by King Henry VII of England, accidentally inaugurated the Fish Revolution; a geo‐economic and cultural phenomenon spanning Newfoundland, Scandinavia, and Western Europe from 1400 to 1700 that transformed the nature of transatlantic politics, from the Hanseatic League in the 15th century, the American Declaration of Independence in the 17th century, to current European Union issues (Holm et al., 2019). As Peter Pope (2012, p. 15) records, in 1502, “Gabriel of Bristol brought home the first recorded cargo of North American cod: thirty‐six tons of salt fish, worth £180 to the merchant Hugh Elyot, an early and persistent transatlantic investor.” This symbolised the first “official” harvest of the Fish Revolution. News of a north‐west Atlantic fishery rapidly diffused through the ports, markets, and trading routes of Europe. Soon, fishing fleets from Portuguese, Basque, and French harbours began long transatlantic tacks to the waters off a “new‐found‐land.” Seasonal voyages across the Atlantic from England to Newfoundland soon developed into a Cod and Sack Triangle, as re‐exporting the species from English ports soon proved inefficient.^2^ As early as 1584, fishing ships were sailing to ports in France, Portugal, and the Spanish Mediterranean directly from the Grand Banks. Wine, raisins, olive oil, and other goods and capital were traded for cod in these Lenten regions and then shipped back to English brokers. This triangle route incorporated the mid‐Atlantic islands of Madeira, the Canaries, and the Azores (Pope, 1996, 2008, 2012). Trade routes between Newfoundland and the Caribbean also supplied lesser grades of cod as a cheap source of salt and protein for the slave diets of the sugar and tobacco plantations, anchoring the Atlantic Slave, Sugar, and Tobacco Triangle. As Pope (2012, p. 80) states:

Newfoundland was neither isolated nor peripheral; it was a central node in an international network. In part, this role followed from the geography of ocean wind and current, which made the Avalon Peninsula a natural watering stop for transatlantic voyagers.

## 2 QUANTIFYING REPRESENTATION: *DISTANT READINGS*, 1500–1830s

**Figure 3:**
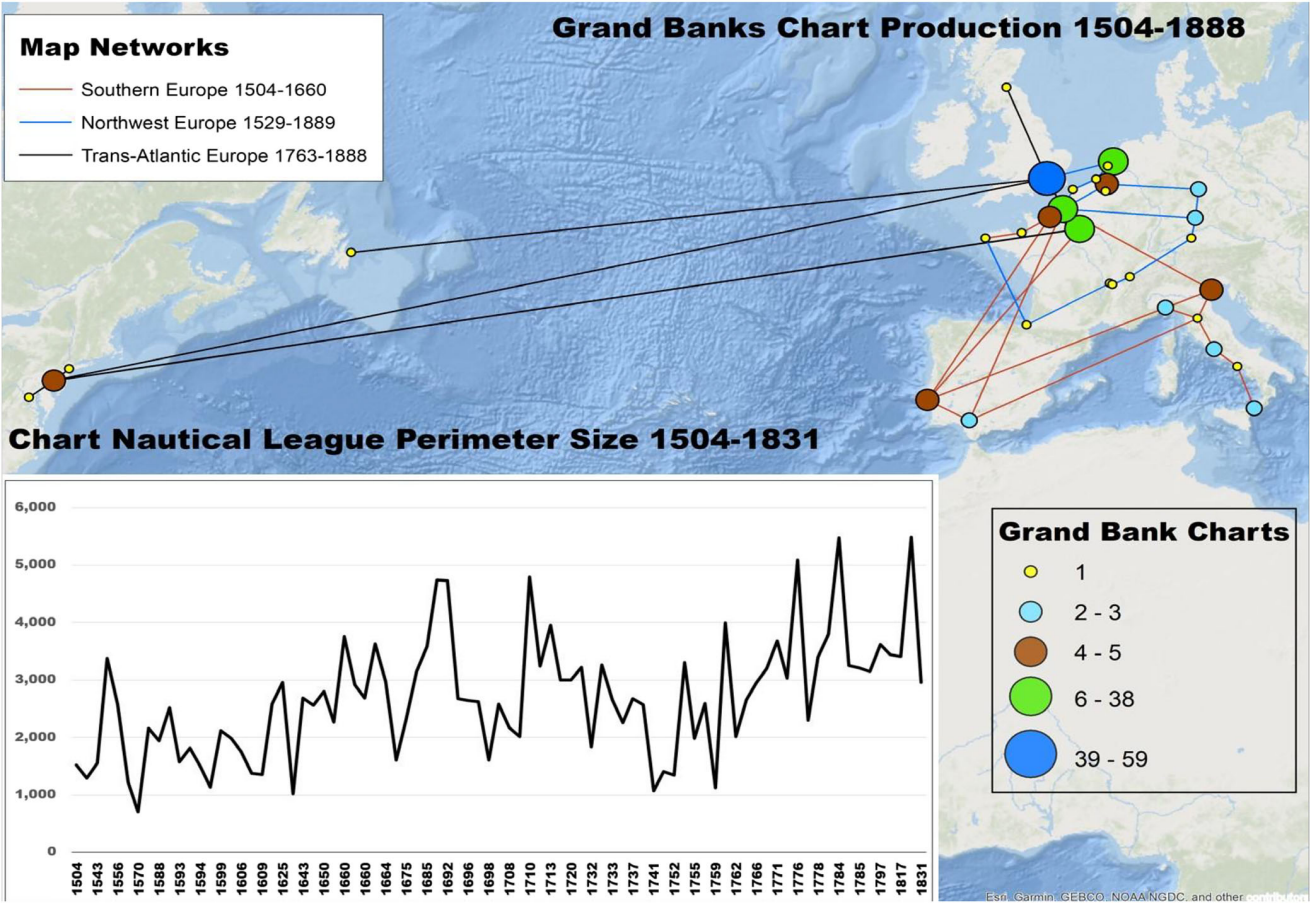
Trans‐Atlantic cartographical constellations. Inset graph: extracted Grand Banks Fishery representation and transformation in size of representation in nautical leagues, from 1504 to 1831 (Charles Travis).

Miles Ogborn (2005, p. 381) notes that Atlantic surveys in geography typically represent maritime environments with “a territorialized map of the continents that border the ocean rather than a chart of the ocean itself. This is rarely maritime history, or an historical geography of the sea.” This paper applies HumGIS *distant reading* methods to correlate archival data on English and French cod catch records between 1500 and 1831, with Grand Bank charts published between 1504 and 1831. Charts of the wider “Newfoundland” maritime environment and its adjacent fisheries grew rapidly in quantity and accuracy from the 14th century to the end of the 19th century. Drafted initially by southern European cartographers (Portuguese, Spanish, Venetian), Grand Banks charts were plotted in increasing numbers from the 16th to the 19th centuries by north‐western European (English, French, Dutch) and colonial American cartographers (Figure 3).

### 2.1 Fishery symbol size and Grand Banks cod catch amounts

A survey of Anglo‐American, Canadian, and French cartographical archives identified 203 Grand Banks charts published between 1504 and 1888 (see Table 1 in Appendix S1) with fishery symbolism consisting of point shading, stippling, dotting, numeral clusters, and other forms of nautical iconography used to signify the extent, area, shallows, shoals, and bathymetric dimensions of the Grand Banks.^3^ Eighty‐three charts dating from 1504 to 1831, with unambiguous symbolism were identified and geo‐rectified in GIS to the current *GSC_WGS_184* geographic coordinate system using Affine transformation methods.^4^ Of the 83 selections, the earliest fishery symbolism appears in the 1504 Portuguese portolan [*Kuntsmann I, Atlantic*] chart by Pedro Reinel. The last fishery symbolism selected was in the American geographer, William Hooker's, 1831 *Chart of the Atlantic Ocean.* Fishery symbolism from 83 geo‐rectified charts was then digitised into polygon shape files and aggregated by area size in measures of both nautical miles and square kilometres. A variable, but clear progression in the size and extent of fishery symbolism in Grand Banks charts published between 1504 and 1831 characterises their morphology over the course of 327 years. This can be interpreted as the result of a growing *ecotonic* relation between increasing numbers of cod harvests, and the roles played by Newfoundland as an inshore fishery and trading hub for the Atlantic Cod and Sack Triangle. To explore the relation between the increasing extent of fishery symbolism and increases in cod catches during the period, Newfoundland port records of English cod catch landings were sourced from Peter Pope (1675–1698) and M. Haines’ (1698–1833) *History of Marine Animal Populations* (HMAP). In addition, Jacquiline Hersart de la Villemarqué’s (1995) *La pêche morutière française de 1550 à 1950* (*The French cod fishery from 1550–1950: statistics, climate, society*) was sourced for French records.

**Figure 4:**
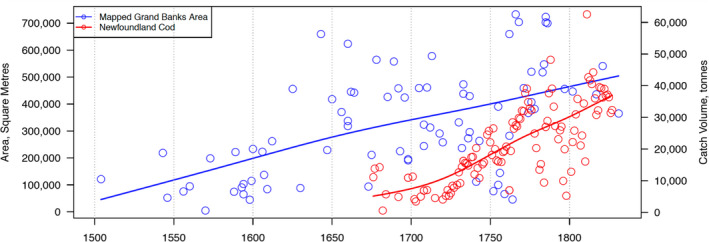
Graph depicts fishery symbolism size of 83 successive Grand Banks charts published between 1504 and 1831 in square metres, correlated with English cod catch in metric tons, 1675–1831. Blue and red lines are exponential trend lines for fishery area and catch volume, respectively (Francis Ludlow and Al Matthews).

**Figure 5:**
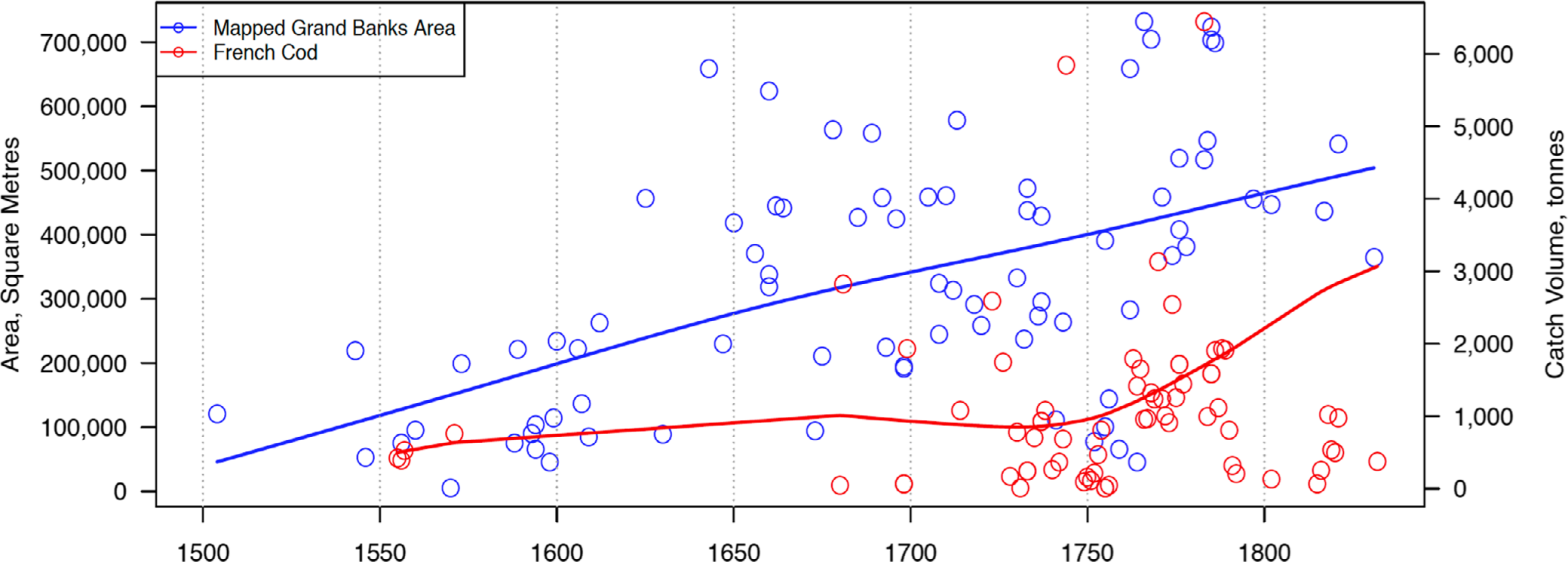
Graph depicts fishery symbolism size of 83 successive Grand Banks charts published between 1504 and 1831 in square metres, correlated with French cod catch in metric tons, 1675–1831. Blue and red lines are exponential trend lines for fishery area and catch volume, respectively (Francis Ludlow and Al Matthews).

**Figure 6:**
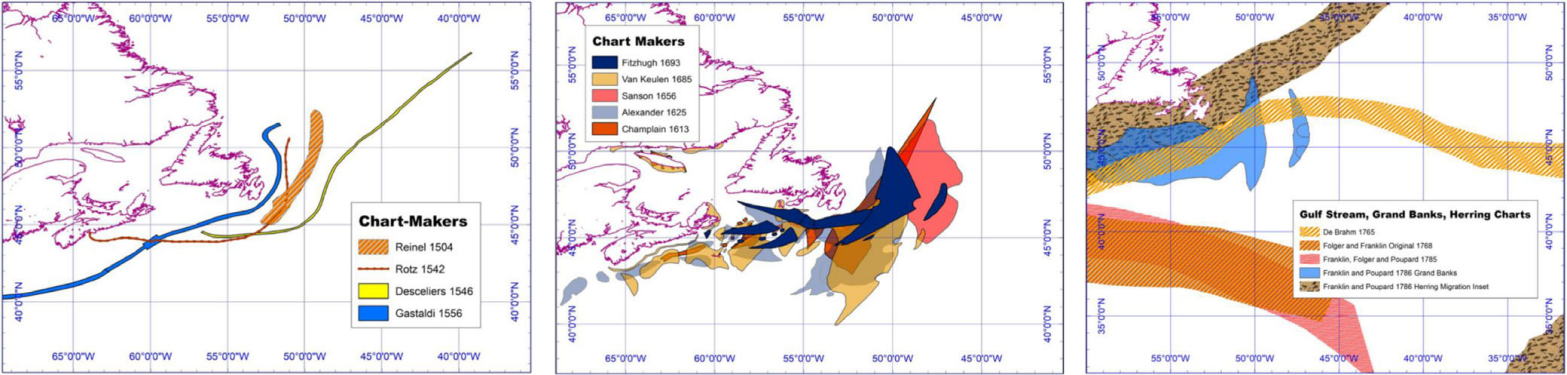
Morphology of Grand Banks symbolism: (a) epistolary traces 1504–1556, (b) Shakespearean fisheries 1600–1700, (c) Atlantic systems 1765–1786 (Charles Travis, Kevin Lougheed, Francis Ludlow, Kieran Rankin).

Correlating Grand Banks fishery symbolism extent by area size and year with the average English catches of each corresponding year (and nine previous years) returned a value of *r* = 0.270.^5^ A correlation test for French catch amounts returned a value of *r* = 0.434. Graphs illustrating increasing catch and fishery symbolism size (Figures 4 and 5) suggest that this is a positive relationship. Although weak to moderate only, the correlations in both cases are consistent in direction (positive) and statistically significant, with a less than 5% chance (*p* = 0.05, one‐tailed) of having occurred randomly (Figure 6).

These correlations suggest an association between the economic growth of Newfoundland fisheries and an evolution in perception, valuation, and prolific charting of the Grand Banks. This correlation does not necessarily indicate cause. Other indirect factors must be considered: technological innovations in navigational, bathymetric cartography, and the invention of the chronometer in 1673 facilitating accurate mappings of standard meridians by the 18th century. In addition, geo‐political scrambles between England and France for the bounty of the “New World” transformed Newfoundland, the Grand Banks, and the New England fisheries into theatres of warfare during the French and Indian (1754–1763) and American Revolutionary (1776–1783) wars. As a result, the waters of the north‐west Atlantic were mapped more extensively. In the next section, the transformation of Grand Banks symbol morphology over the *longue durée* of the Fish Revolution is parsed with HumGIS into a triptych of Braudelian historical cycles: *traces* (1504–1556), *fisheries* (1600–1700), and *systems* (1765–1786).

## 3 QUALIFYING PERCEPTIONS: *CLOSE READINGS*, 1504–ca 1787

Under the focus of a HumGIS lens, a *close reading* of chart symbolism reveals a three‐cycle morphology of the Grand Banks cartographic gaze that emerged between 1500 and 1800. First, as navigational line *traces* (1504–1556), parsed with epistolary accounts of John Cabot's iconic 1497 voyage. Second, as a geographical and textual hermeneutic on nationalism, colonialism, and commerce through the charting of *fisheries* (1600–1700) and the Shakespearean drama *The Tempest* (1611). Third, the early surveys on the Gulf Stream and mechanics of oceanic *systems* (1765–1786) in DeBrahm's *The Atlantic Pilot* (1772) and Franklin's *Sundry Maritime Observation* (1785) – understandings that emerged amidst the furore of early modern scientific and republican revolutions (see figure 6).

## 4 TRACES (1504–1556): EPISTOLARY CARTOGRAPHIES

**Figure 7:**
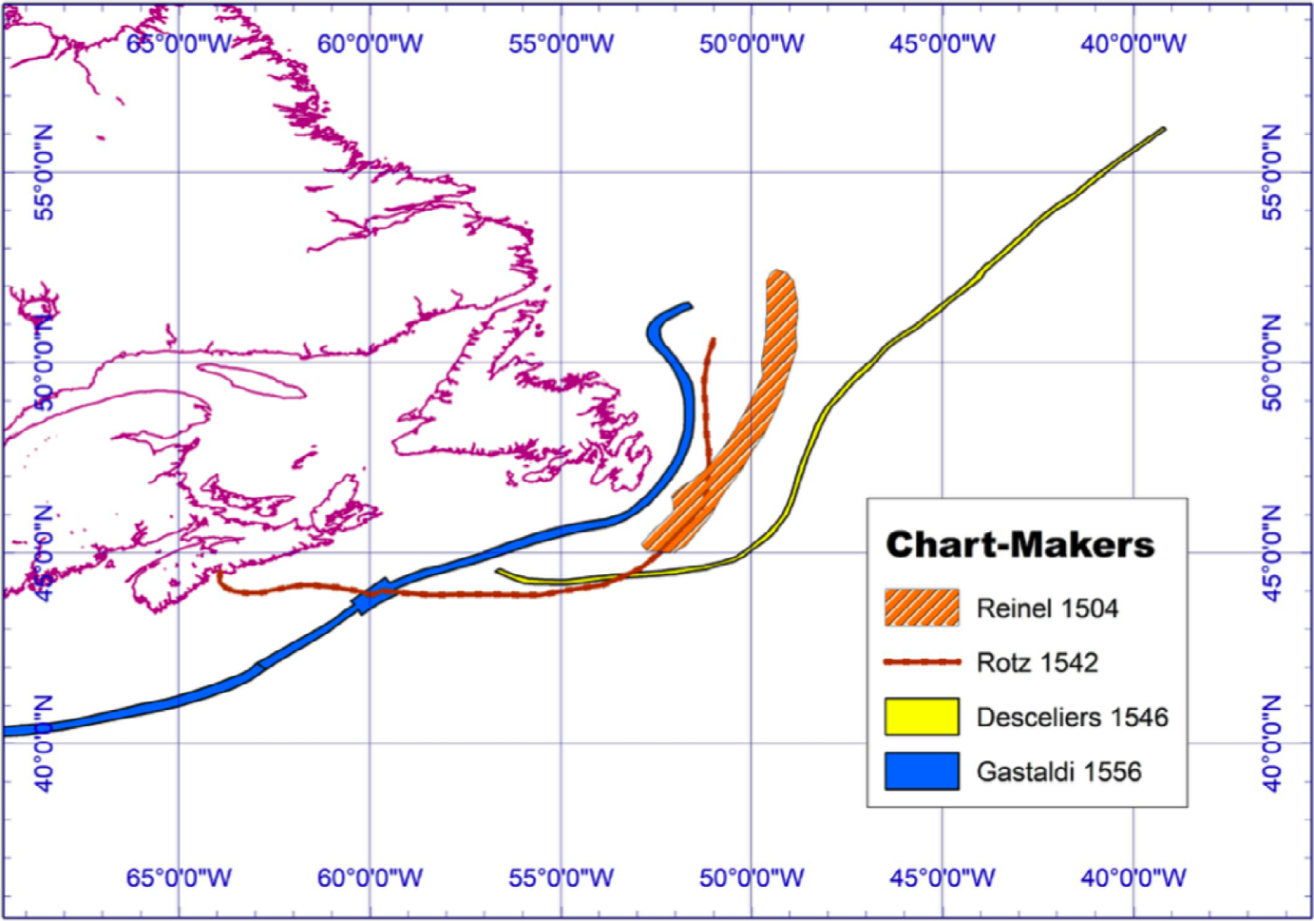
(A) Traces, 1504‐1556, Grand Banks symbolism of Pedro Reinel (1504) Jean Rotz (1542) Pierre Desceliers (1546) and Giambattista Gastaldi (1556) (Charles Travis, Kevin Lougheed, Kieran Rankin).

In the 15th century, at the time of Cabot's voyage, the most prominent works of academic geography, based upon the rediscovery of Ptolemy's work, bore little relation to the real experiences of navigators on the high seas. For sailors, the cartography of the period was a “paralyzing discouragement” to explore the Western ocean, with the various redrafted Ptolemaic *mappae‐mundii* practically useless for the purposes of actual navigation (Parry, 1969). However, 15th century sailors did not go to sea without charts. From the 13th century at least, in the Italian and Catalan ports there existed a school of professional hydrographers who drew *portlani* charts intended for use at sea, based on sailing experiences and owing little to the formal academic sciences of the period (see figure 7).

The first *portolani* tracings (1504–1556) appear more like scribbles on a piece of parchment, approximate period navigational lines rather than the actual extent of the Grand Banks fisheries. As ships sailed to North America from Europe, navigators plotted by dead reckoning due to increases in magnetic deviation from true North, leading to routes that inclined farther west. Such error‐prone methods were compounded by difficulties in calculating longitude and led to speculative charts. This navigational uncertainty provides a rationale for arguing that Cabot did not discover or actually land in Newfoundland in June 1497. Initially, Cabot, thinking he had discovered the mythical *Island of the Seven Cities*, assigned the southernmost part of his landfall latitude 45° north, commensurate with Bordeaux's Gironde Estuary, and corresponding to a modern‐day location in Nova Scotia, east of Halifax. No charts were drafted of Cabot's iconic voyage. However, on his return to Bristol, two letters providing contradictory navigational accounts of the journey were dispatched by observant eyes to Seville, Spain and Milan, Italy.

### 4.1 Day and Pasqualigo letters 1497–1498

Keighren and Withers (2011, p. 1332) note that “correspondence is a matter of … an epistemic *desideratum*: That what is written about should correspond in some way to the thus described.” The *John Day Letter of 1497–1498* and the *Lorenzo Pasqualigo Letters of 23 and 24 August 1497* respectively convey contrasting sea‐log and navigational data. In addition, the geographical features sighted during the voyage seem to incorporate a coastal panorama well beyond the Newfoundland shoreline. After re‐examining the letters, a small cohort of modern scholars argue that Cabot never actually landed on the island, nor was he the first to travel to the Grand Banks fishery in the late medieval period (Jackson, 1963; Jones & Condon, 2016; Quinn, 1961; Ruddock, 1966; Sauer, 1968; Vigneras, 1956; Williamson, 1962). After an unsuccessful voyage in 1496, Cabot set out again from Bristol, England on 20 May 1497; approximately five weeks later *The Matthew* sighted what was claimed to be Newfoundland (Williamson, 1962). Cabot claimed he followed the coast eastward for about a month, before turning, and returning to European shores in 15 days, landing first at Brittany, due to a navigational error, before sailing to *The Matthew's* home port of Bristol. The *Pasqualigo Letters* contradict themselves on the distance Cabot and his crew travelled before landing: *23 August* – 700 leagues (3,889 km) and *24 August* – 400 leagues (2,222 km). The *John Day Letter* states that the cape nearest to Ireland that Cabot sighted was located 1,800 miles (2,896 km) west of Durnsey Head, Ireland.

It has also been argued that Cabot may have first sighted land on North Cape Breton after which the expedition sailed south to about 45° (South Cape Breton) and then double backed northward as far as 51° 30' (Jackson, 1963; Quinn, 1961; Ruddock, 1966; Sauer, 1968; Williamson, 1962). It seems this “nearest” cape may have been discovered when Cabot reversed course to begin the return journey, and it is speculated that it is from this location that *The Matthew* sailed for Bristol. Therefore, the land potentially explored by Cabot ranged from 45° to 51° 30'; in other words, from central Nova Scotia to the northern tip of Newfoundland, or possibly along the New Brunswick coast and then along the western shore of Newfoundland to the mouth of the St Lawrence Seaway. Cabot recorded that the needle of *The Matthew's* compass “north‐wested,” introducing a 2° error in locational fixes (Jackson, 1963).

**Figure 8:**
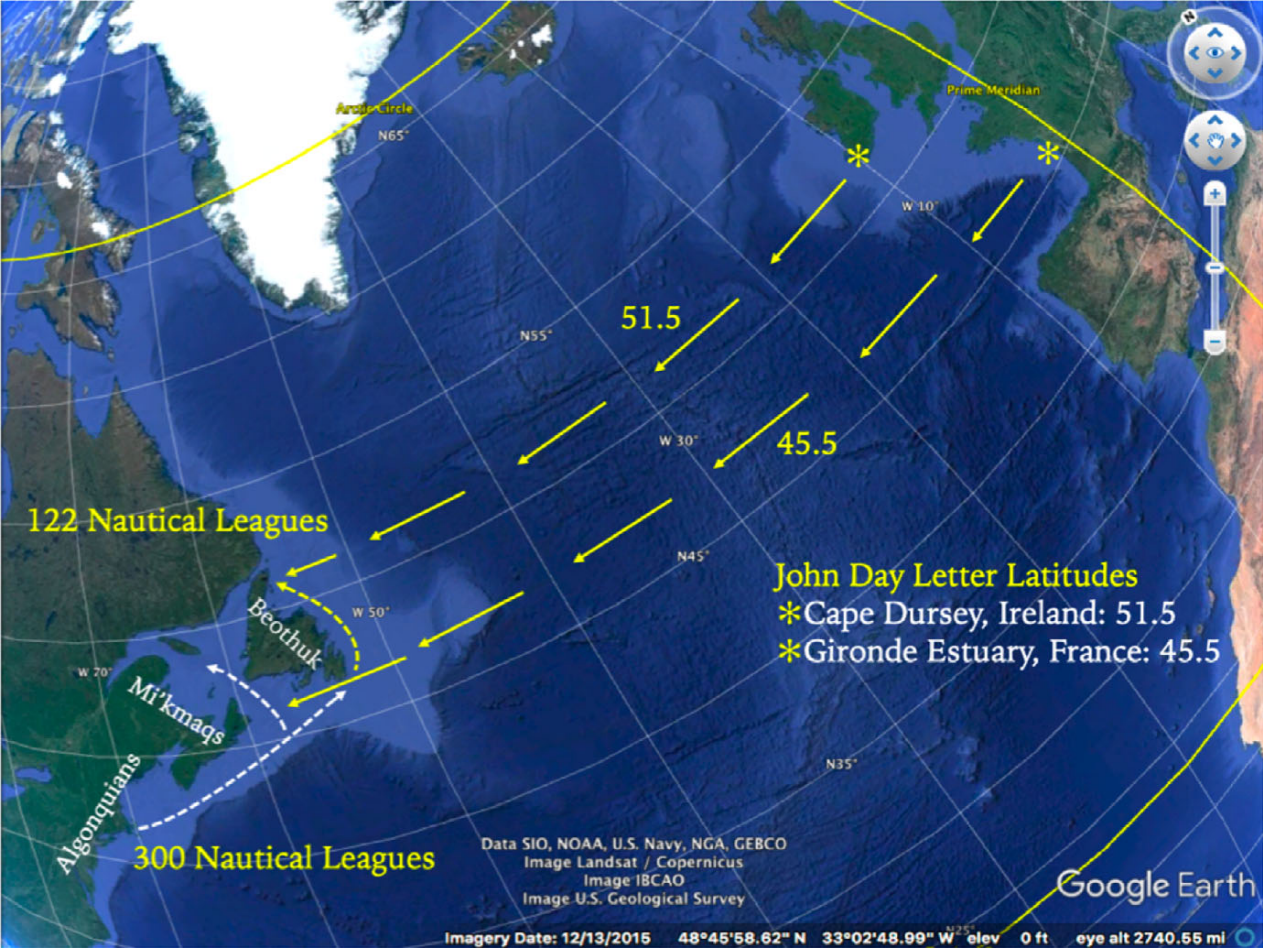
Speculative, Epistolary Mappings: Lorenzo Pasqualigo 1497 and John Day 1497‐98 Letters (Charles Travis).

Furthermore, the distance Cabot travelled to the “new found land,” recorded in both the *Day* and *Pasqualigo Letters*, falls short compared with a measure of the actual distance of 4,817 km. This could be attributed to faulty recollections, mistranscription, or errors in distance record keeping. It is apt to note that *The Matthew* landed in Brittany on its return leg because the crew informed Cabot that he was sailing at a latitude too northwardly to reach Bristol; by over compensating, his ship erroneously landed too far south. The 23 August 1497 *Pasqualigo Letter* describes Cabot coasting the north‐west Atlantic shoreline for 300 leagues (1,600 km) (see Figure 8).

Though this coasting distance could also be erroneous (if taken at face value, it implies that if Cabot did land on the eastern shore of Newfoundland, he would have coasted up well past the shoreline of present‐day Labrador) given that the entire coast of eastern Newfoundland would have been coasted in approximately 122 leagues, giving rise to an argument that Cabot actually landed in Labrador (Jackson, 1963). Yet, there is no mention of any iceberg flows, which even in June and July would have been likely present this far northerly. Taking again the 300‐league coasting measurement at face value, and read in juxtaposition with human and natural landscape descriptions from the *John Day Letter*, it has been observed that the latitudes 45.5° north and 51.5° (paralleling the Gironde estuary in France, and Dunsey Head in Ireland), which Cabot coasted in the north‐west Atlantic, are too northward to match the geographical features described in the *Day Letter*:

… they found large trees of which masts of ships are made … and a land of much herbage … they saw cultivated fields where they thought people lived … They went on about a month discovering the coast, and from the above‐mentioned cape of the mainland that is nearest to Ireland they returned to the coast of Europe in fifteen days with a following wind, landing in Brittany because the seamen distracted him by saying that he was headed too far to the north … (Sauer, 1968, p. 34)

Given this description, the possibility of Cabot's landing on eastern Newfoundland's rocky and peat‐covered Atlantic coast becomes remote. Provided with an account of “cultivated fields” it is not too much of a hazard to conjecture that Cabot's landing occurred between Nova Scotia and Cape Breton; the indigenous Beothuk of Newfoundland were not large‐scale agriculturalists. The fields described may have been those of the Algonquian tribe (Figure 7) indigenous to modern New England (Sauer, 1968). But if the landing was further south‐eastward in Nova Scotia, it could be speculated that *The Matthew* rounded Cape Breton and tacked eastward into the Gulf of St Lawrence. Cabot then coasted along the shorelines of Prince Edward Island and New Brunswick, observing the fields of the Mi'kmaq tribes, before westing, and slipping unknowingly into the Gulf Stream. The stream's current, along with the strong prevailing westerlies, could explain the swift 15‐day return voyage to Bristol (as opposed to the five‐week outbound leg), from which the *Day* and *Pasqualigo Letters* were dispatched.

## 5 FISHERIES (1600–1700*)*: *O BRAVE NEW WORLD*

… but Nature should bring forth,

Of its own kind, all foison, all abundance,

To feed my innocent people.

(Folger Shakespeare Library, 2018, pp. 178–180)

**Figure 9:**
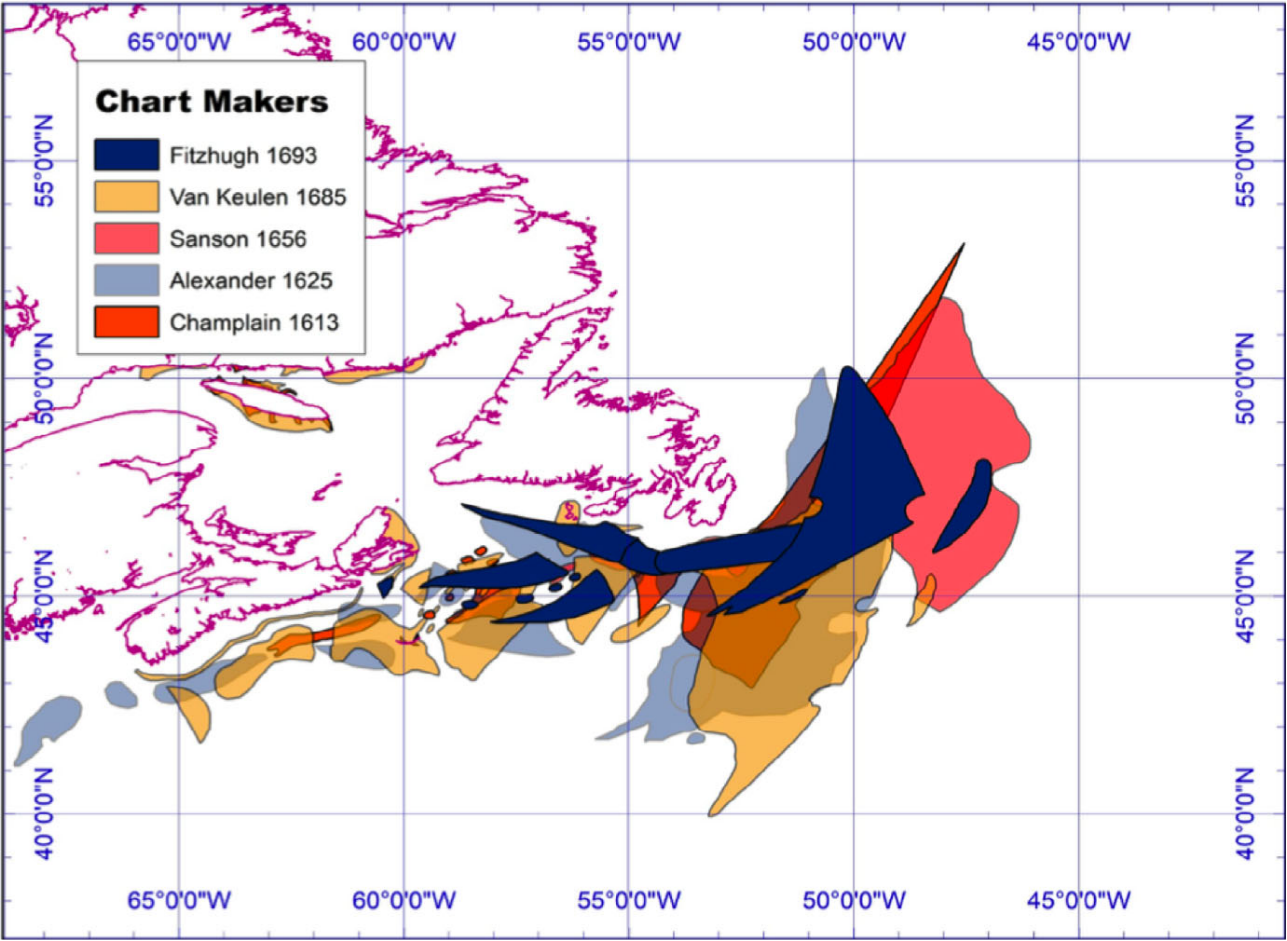
Fisheries 1600‐1700 (Charles Travis, Kevin Lougheed, Kieran Rankin). [Correction added on 2 May 2020, after first online publication: The source notes originally appearing under Figure 9 have been deleted as they were not meant for this figure.]

In August 1497, the Duke of Milan received a letter from Lorenzo Pasqualigo, his ambassador to England. In the correspondence he learned news of Cabot's return from a “newfoundland” with waters “swarming with fish, which can be taken not only with the net, but in baskets let down with a stone” (Lawrence & Young, 1931, p. 274). And it is the fictionalised Duke of Milan, who features prominently in the Shakespearean island‐bound drama, *The Tempest* (1611). First performed for King James on *Hallowmas nyght*, 1 November 1611, in the candlelit space of the Blackfriars Theatre in London, *The Tempest* tells the story of Prospero, the rightful Duke of Milan, who along with his daughter Miranda, is exiled on a mysterious isle after being ousted by his brother Antonio from a duchy. Many Shakespearean dramas possess conflicts with a maritime dimension and “the sea (including its fish and storms), ships, voyaging, and sailing” play literal and figurative roles as their acts unfold (Cohen, 2000, p. 158) (see Figure 9).

Scene 1 of *The Tempest* opens to the “tempestuous noise of thunder and lightning heard” on a ship's deck surrounded by “the cheeky wind blowers of sixteenth and seventeenth century world maps” (Brayton, 2012, p. 180). Prospero is attended to by two servants: Ariel, a spritely spirit, and the indigenous Caliban, who is rendered as “legged like a man, and his fins like arms!” (Act 1, Scene 2, pp. 34–35). Prospero orchestrates a storm with his alchemical powers, causing the shipwreck of his brother Antonio, Alonso the King of Naples, and his son Ferdinand who are sailing in return from the wedding of the king's daughter, Claribel, in Tunis. Survivors emerge from the sea and straggle their way to Prospero's abode, where Fernando is imprisoned. A fanciful assassination plot is devised by Caliban after being discovered among flotsam and jetsam on the island's beach by the jester Trinculo and drunken butler Stephano, who escaped the wreck by a “butt of sack which the sailor's heaved o'erboard” (Act 2, Scene 2, pp. 125–126). With the aid of Ariel, Prospero thwarts the plot, conjuring up a series of magical encounters between *The Tempest's* characters, which leads to the marriage of Miranda and Ferdinand, and the restoration of Prospero's dukedom (Figure 8).

It can be argued that the drama is linked to the rise of oceanic English nationalism, its navy, the first modern plotting of the Grand Bank and its fisheries, and the development of cod markets and international commerce in Shakespearean London (Figure 9). *The Tempest* also illustrates the accumulation of maritime knowledge from Spanish, Portuguese, and Italian navigators facilitated by Henry VIII’s establishment of English nautical cartography at Trinity House in 1514: “like a palimpsest holding the cultural memory of five centuries” the play preserves “the many traditions of the sea” (Sobecki, 2008, p. 165).

Sir Humphrey Gilbert declared English possession of Newfoundland in 1583 at St John's Harbour, before a fleet of over 30 fishing ships from Spain, Portugal, France, and England. In *The Tempest* the claim on the island's fishery and new source of English wealth may been seen reflected in the lines:

GONZALO

Had I plantation of this isle, my lord‐ …

I’ th’ commonwealth I would by contraries

Execute all things, for no kind of traffic

Would I admit; no name of magistrate;

…

I would with such perfection govern, sir,

T’ excel the Golden Age.

*The Tempest* (Act 2, Scene 1, pp. 157–184)

During the late 16th and early 17th centuries, Newfoundland's fisheries were viewed as more of an extension of the three Kingdoms of Britain, than as a region of the New World. More than 33% of the MPs in the English Parliament between 1604 and 1620 invested in transatlantic trading enterprises, such as the Grand Banks Sack and Cod trade (Cohen, 2000). English merchants came to dominate the lucrative salt‐dry codfish trade with the Lenten Catholic countries of France, Spain, and Italy. Soon Newfoundland which was not “isolated or peripheral” became “a new colonial model for England” due to the “geography of ocean wind and current” (Pope, 2012, p. 80; Test, 2008, p. 202).

In contrast to economic losses incurred at Jamestown, and the failed colony at Roanoke, Newfoundland housed a fluid, mobile labour force that produced New World profits from Old World trade in Poor John [cod], and a “nursery of seamen” for its navy to counter continental European interests in the region (Test, 2008). Indeed, early‐modern transatlantic trade, as well as the slave diets of the Caribbean plantations, depended upon this food staple given that it was nearly impossible for any ship to sail to the West Indies without Newfoundland cod. No other species could “endure to pass the line sound and untainted, but the fish of that country salted and dried there” (Innis, 1971, p. 52). Shakespearian studies of *The Tempest* have focused on the influences of English naval activity in the Mediterranean, the Caribbean, Bermuda, and Virginia Colony, on the Shakespearean rendering of the play's mysterious isle (Brotton, 2004; Fuchs, 1997; Kermode, 1980; Mentz, 2009; Mulready, 2013; Nosworthy, 1948; Wylie, 2000). In particular, William Strachey's story of the wreck of *Sea Venture* in 1609 near Bermuda was thought to be a direct influence. However, scholars now look to English entanglements in the Ottoman Empire, Ireland, and most recently the north‐west Atlantic oceanic plantation. The “deep nook,” one of the features in the Shakespearean collage depicting the island in *The Tempest*, could certainly be attributed to Newfoundland's fjords:

ARIEL

Safely in harbour

Is the King’s ship; in the deep nook, where once

Thou call’dst me up at midnight to fetch dew

From the still‐vexed Bermoothes, there she's hid;

*The Tempest* (Act 1, Scene 2, p. 269)

**Figure 10:**
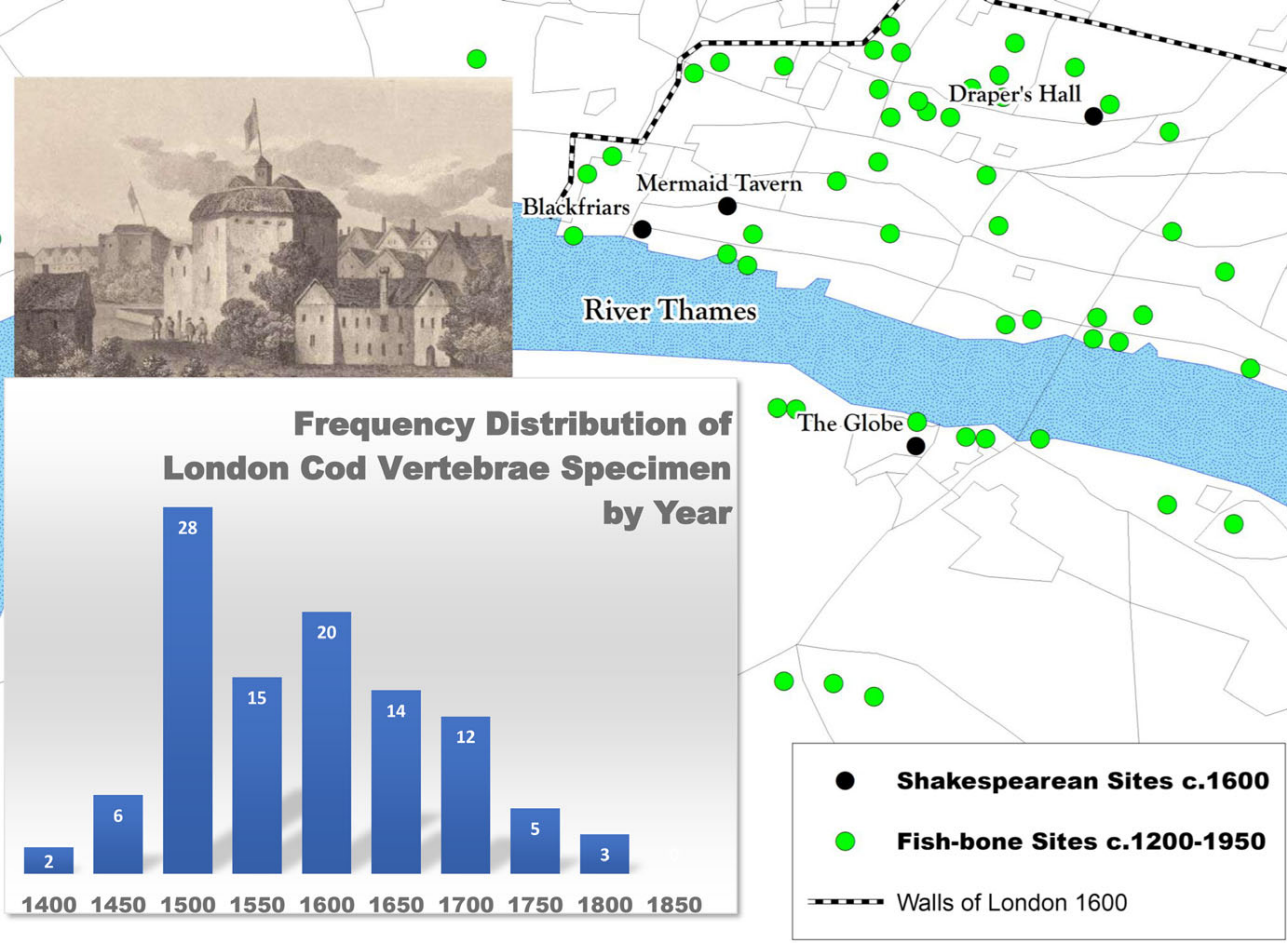
Location of London sites included in this study. *Source*: Orton, D.C., Morris, J., Locker, A. and Barrett, J.H., (2014) Fish for the city: meta‐analysis of archaeological cod remains and the growth of London’s northern trade. Antiquity, 88(340): 516‐530. Inset Detail of The Globe Theatre, Southwark, London, England c. 1612. After the engraving from Wilkinson’s Theatrum Illustrata (1825) (Map image and graph by Charles Travis)

Sable Island, south‐west of the Grand Banks, called the “Graveyard of the Atlantic” (Cameron, 1965, p. 463) because of the high number of ships wrecked on its shoals, could also be a source of inspiration. Because of its shifting shorelines, the island is treacherous to navigate in fair or foul weather. The “Graveyard of the Atlantic” is symbolised in Giacomo Gastaldi's 1556 chart as a rectangular box (Figure 10) off the coast of Nova Scotia labelled *Isola dell arena* (Isle of Sand). Sir Humphrey Gilbert's flagship, the *Delight*, grounded on Sable Island in 1583 with the loss of 93 souls after claiming Newfoundland for England (Cameron, 1965; Pope, 2012; Probasco, 2014). In 1610, a year before the premiere of *The Tempest*, the London and Bristol based Newfoundland Company underwrote the first English fishery settlement in Cupids Cove. Caliban's indigenous claim on the island can be read as an allusion to English colonial encounters with Newfoundland's indigenous Beothuk people. But also, as Prospero informs Miranda, Caliban “serves in offices/That profit us” (Act 1, Scene 2, pp. 374–375). The claim could be read as an allusion to the fishery labour and settler pool drawn from the districts near the Thames, the west of England, and Ireland, which created wealth for the Newfoundland Company:

CALIBAN

This island’s mine by Sycorax, my mother,

Which thou tak’st from me. When thou cam’st first

…

And showed thee all the qualities o’ th’ isle,

The fresh springs, brine pits, barren place and fertile.

… Which first was mine own king; and here you sty me

In this hard rock, whiles you do keep from me

The rest o’ th’ island.

*The Tempest* (Act 1, Scene 2, pp. 396–405; 409–411)

Matthew Test (2008) argues that studies discussing the geographical and socio‐cultural‐political and economic influences on *The Tempest* neglect the hub of shipping, trade, and consumption on the River Thames relating to Grand Banks fishery commercial ventures in London, where The Globe and Blackfriars theatres were located: “it would have been impossible to cross London Bridge or work in Southwark without seeing galleys, galleons, barges, wherries, pinks, hoys, cogs and all manner of fishing and trading craft on the river” (Brayton, 2010, p. 92). Located on the north side of the River Thames, The Blackfriars was situated in an atmosphere pungent with the language of fishmongers and sailors drinking close by at the Mermaid Tavern. The Globe, established in 1599, was located in the bull and bear baiting, brothel, and gaming district of Southwark, outside London's city walls on the Thames. C.J. Visscher's 1616 cityscape of London graphically depicts the “South Warke” locations of the “The Bear Garden” and “The Globe” (incorrectly labelled in reverse) with a cavalcade of sailing vessels in the *Thamesis Fluvius* background (see Figure 10).

The Shakespearean theatres occupied a landscape of London scattered with archaeological fish‐bone sites dated by Orton et al. (2014) to between 1200 and 1900. Once the locations of fish markets, taverns, and waste dumps, the sites were identified as places where cod was consumed and their bones discarded. As the graph in Figure 9 illustrates, in 1500 there was an explosive leap in cod consumption (based upon frequency distributions of vertebrae finds at London fish‐market sites) before dipping and stabilising in the 1600s (Orton et al., 2014). In Act 2 of *The Tempest*, Caliban is described seemingly as half‐human and half‐cod. The mention of silver perhaps alludes to the lucrative cod‐fish trade occurring near The Globe and Blackfriar theatres:

TRINCULO

What have we here, a man or a fish? Dead or

alive? A fish, he smells like a fish ‐a very ancient

and fishlike smell, a kind of not‐of‐the‐newest poor‐John.

A strange fish. Were I in England now,

… but would give a piece of silver.

*The Tempest* (Act 2, Scene 2, pp. 24–30)

**Figure 11:**
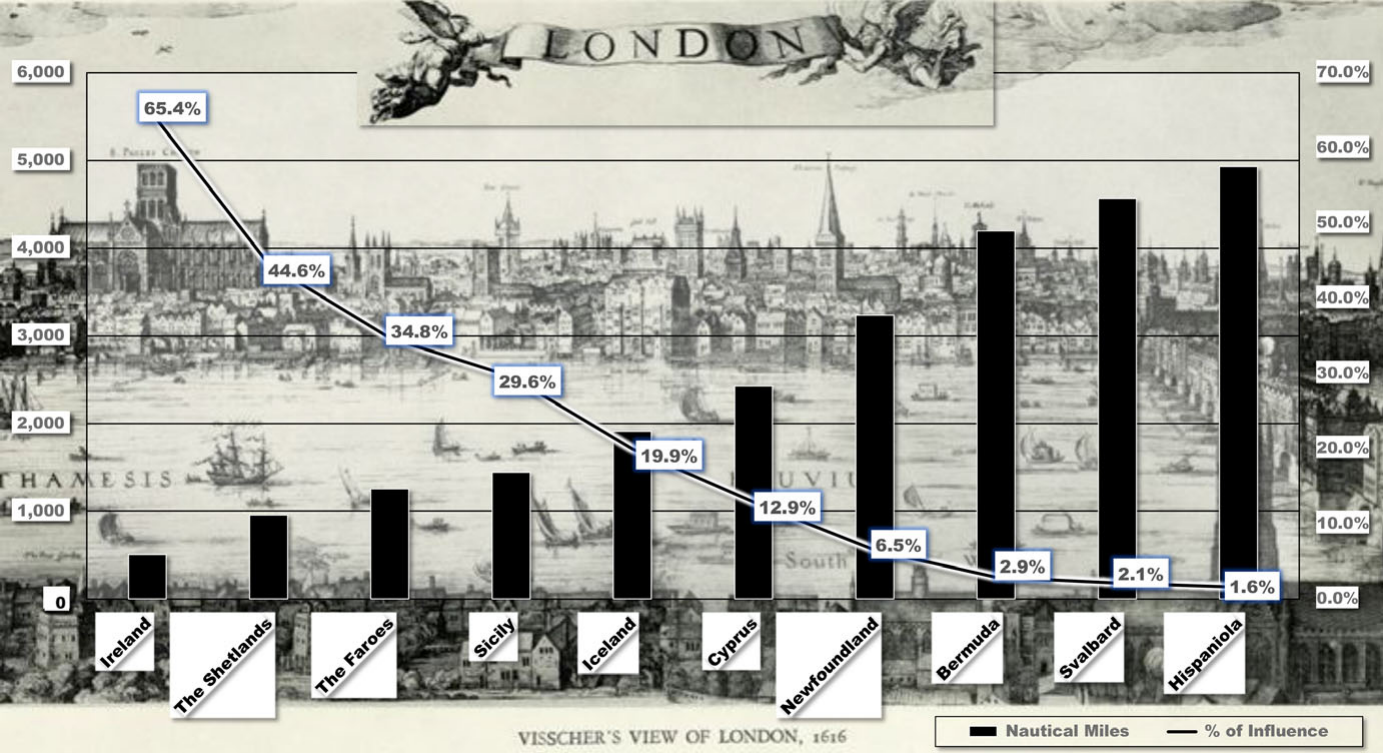
*The Tempest* distance decay. Graph: distance decay of possible locational influences on the island setting of *The Tempest* from site of The Globe theatre (left: nautical miles; right: % of influence). Upper panel: C.J. Visscher's 1616 Cityscape of London (Al Matthews and Charles Travis).

Indeed, it can be argued that Shakespearean theatres and their environs influenced the setting of *The Tempest* as much as any of the Mediterranean or Atlantic islands and coasts anchoring the webs of English oceanic expansion. Shakespeare's contemporary, the playwright Ben Jonson, called the London brothel district “the Bermudas,” described in *The Tempest* as the “still‐vexed Bermoothes” (Act 1, Scene 2, p. 272; Skura, 1989, p. 59). Meridith Anne Skura (1989, p. 53) argues “there is still a strong need for research that moves beyond *The Tempest* to consider Shakespeare's drama within a larger network of global trade.” (see Figure 11)

Taking The Globe and Blackfriars theatres (opposite the Thames from each other) and their fish‐market and port milieu as ground zero, a *distance decay* mapping of possible island locations, as well as commercial influences on the setting of *The Tempest* was conducted. As Tobler (1970, p. 236) observes, the “first law of geography” is that “everything is related to everything else, but near things are more related than distant things.” Distance decay measures interactions between two locales in terms of bio‐geographic, social‐cultural, economic, political, and any other phenomena that can be identified. Also known as the *inverse distance effect*, its analysis plots “the lessening in force of a phenomenon or interaction with increasing distance from the location of maximum intensity” (Oxford Dictionary of Geography, 2015, p. 139). In other words, it posits that a variable's influence at one location declines as a factor of influence as the distance between it and other locations increase. Postmodern compressions of distance, time, and space facilitated by 20th century broadcast media and the worldwide web seems to relegate distance decay analysis to the geographical curio shop. However, Shakespearean period spatio‐temporal sensibilities preceded the clockwork universe and Einsteinian perspectives of time and space.

As Waldo Tobler (1970, p. 234) states, it is with “ceterus paribus,” that distance decay analysis can serve as a tool to speculate on locational influences on a drama written and performed in the centre of a world port coursing with international trade and correspondence. As has been noted, Strachey’s 1610 account of a 1609 Bermuda shipwreck has been speculated as a major influence on *The Tempest*. However, through the lens of a distance decay analysis (Figure 11), Bermuda's location returns a value of 2.9% of influence – notably less than the Mediterranean locations of Cyprus at 12.9% and Sicily at 29.6%. In the north Atlantic, a region with many English colonial and commercial ventures, locational influences returned a 65.4% value for Ireland and a value of 6.5% for Newfoundland, but over twice the influence value of Bermuda. Values were estimated with a negative exponential decay curve in which an exploratory baseline value of 90% was chosen to represent the level of decay at 125 nautical miles from the origin or central point, in this case the location of The Globe Theatre, which was assigned a value of 100%. The decay of influence persisted asymptotically with increasing distance, but never reaching 0% (or, put differently, doing so only at infinite distance from the location of The Globe). Influence halved to 50% at 822 nautical miles, representing a “half‐life”; each additional distance of 822 nautical miles represented a further halving (i.e., 50%, 25%, 12.5%, and so on). Using this approach it can also be speculated that locations in the North Atlantic closer to London's latitude of 51.5°N (Newfoundland 6.5%, and Ireland 65.4%) have a 54% average influence, higher than locations notably south of London's latitude (Cyprus 12.9%, Sicily 29.6%, and Bermuda 2.9%), which possess a 15% collective average influence. As Jerry Brotton asserts:

the play is precisely situated at the *geopolitical bifurcation* between the Old World and the New, at the point at which the English realized both the compromised and subordinated position within which they found themselves in the Mediterranean, and the possibility of pursuing a significantly different commercial and maritime initiative in the Americas (2004, p. 37).

Although certainly exploratory, perhaps fanciful and statistically speculative in nature, this distance decay analysis does suggest that 17th century English colonial and fishery ventures in the north Atlantic should be examined more closely in Shakespearean studies.

MIRANDA

O wonder!

How many goodly creatures are there here!

How beauteous mankind is! O, brave new world,

*The Tempest* (Act 5, Scene 1, pp. 215–219)

**Figure 12:**
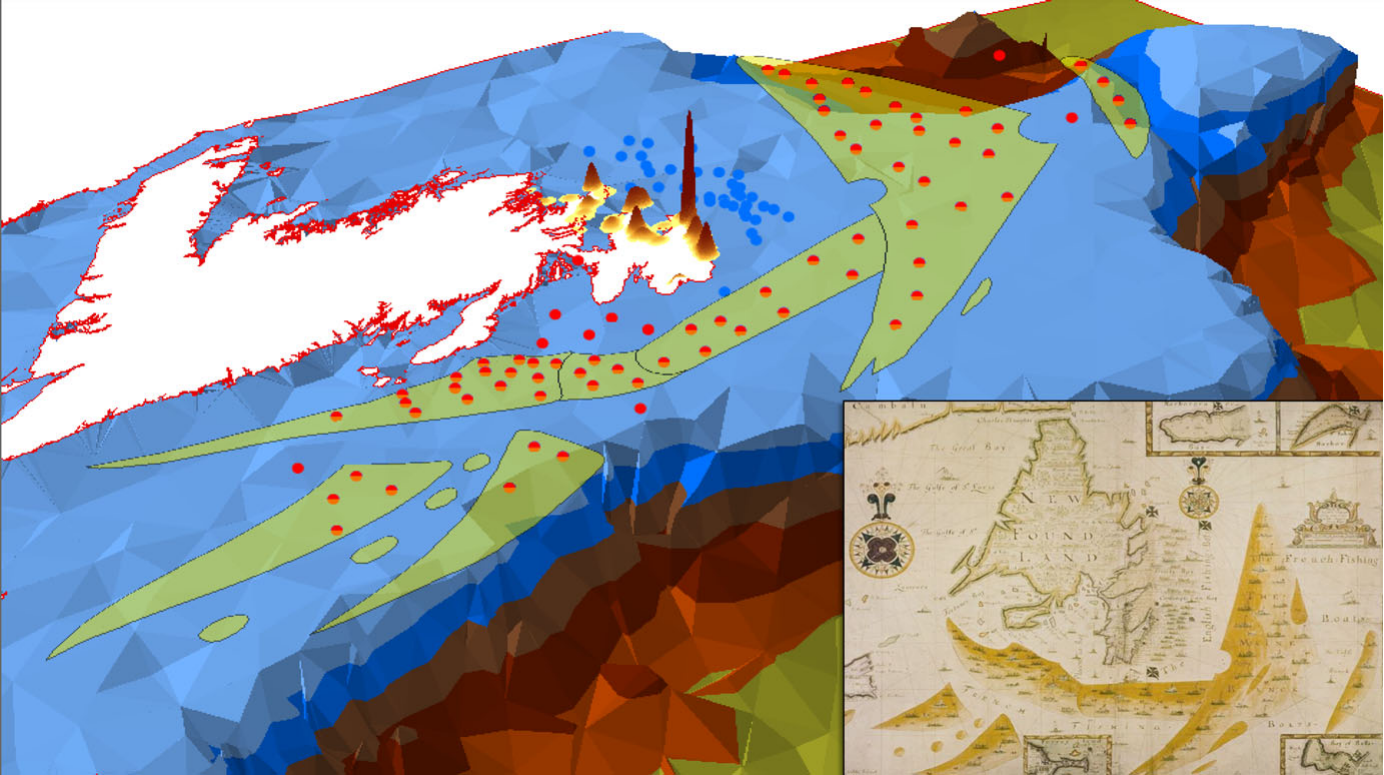
“A mountain hid under water.” Inset chart: Augustine Fitzhughe (1693), a chart of the coasts of Newfoundland, with the fishing districts marked (1693, London), 122 × 69 cm (© Board of the British Library – Additional MS. 5,414.30). Deformed chart: English boats (blue dots) and French boats (bed dots), 3D kernel density of English 1675–1698 catch per Newfoundland port (Peter Pope HMAP Data) NOAA/Canadian Bathymetric Data (Charles Travis).

The Shakespearean late canon sat on the cusp of the scientific revolution. The body of work it represents has not only been described as an accretion of period geographical, cartographical, and maritime knowledge, but also an influence on the theatre space of The Globe (Cohen, 2006; Rampling, 2014). The Thames School of Cartography (1590–1720), occupying the Shakespearean and fish‐bone landscapes of London, may have also been an influence. Concentrated along the Thames, the school's drafting houses, like theatres and cod markets, were cheek by jowl with the milieu of ships, mariners, and merchants; their charts (or *platts*) clearly expressed English commercial and colonial interests (Maeer, 2006; Smith, 1968). Institutionalised by membership in the Drapers’ Company of London, its membership included copyists and cartographers that produced portolan style charts, called platts (often copies of Dutch nautical cartography). The school participated in England's propaganda war over the threat of French fishing fleets to its Newfoundland oceanic plantation. A striking example is *A chart of the coasts of Newfoundland, with the fishing districts marked*, drafted in 1693 by platt‐maker Augustine Fitzhugh (Figure 12).

Adorned by two elaborate compass‐roses, “Fitzhugh's chart outwardly politicizes the fishery by differentiating the French and English fishing fleets (with flags dotting the map)” by plotting “how a smaller English fishing fleet is being surrounded,” conveying the impression that French offshore fleets are encroaching on inshore English fishing waters (Maeer, 2006, p. 192). Cartographic symbolism from the 1693 chart, superimposed upon a HumGIS bathymetric model, is juxtaposed by 3D kernel density extrusions of Newfoundland English port cod catch landings recorded between 1675 and 1698.^6^ Fitzhugh's propaganda chart preceded Queen Anne's War (1702–1713), itself a theatre of the Spanish War of Succession (1701–1714). The 1693 chart presages an anonymous staging of the revised *The Tempest of Enchanted Island* as the Seven Years’ War (1756–1763) was reaching its conclusion (Taylor, 2012). The restored Duke of Milan's proclamation as the curtains close possesses a prophetic resonance:

PROSPERO

… I have bedimmed

The noontide sun, called forth the mutinous winds,

And ’twixt the green sea and the azured vault

Set roaring war; to the dread‐ rattling thunder

Have I given fire

*The Tempest* (Act 5, Scene 1, pp. 50–55)

In June 1762 the French captured St John's, Newfoundland, burning English vessels and fishery infrastructure. However, coinciding with *The Tempest's* restaging in September, the British counter‐attacked, bringing its “roaring war” and “dread‐rattling thunder” to life in recapturing Newfoundland. It has been argued that both as text and performance, *The Tempest* “is a prologue to the whole thrust of technological modernity,” that emerged with the “the modern world‐system” to become one and the same (Bate, 1991, p. 77).

## 6 SYSTEMS (1765–1786): THE “GULPH STREAM”

By the 18th century, knowledge had emerged of oceanic gyre dynamics and currents of the Gulf Stream, due to their effects on shipping times. It is clear today that the Gulf Stream and the *Derive Nord Atlantique* (DNA) constitute currents that drive the earth's climate and the abiotic environment of the Grand Banks fishery that anchored the Cod and Sack Triangle. As Pope (2012, pp. 80, 158) notes

… the trade in salt cod was part of a complex commercial web that linked Newfoundland not only with the West Country but also with London, Iberia, the Mediterranean, the Atlantic islands, the Netherlands, New England, and even New France; [indeed] this triangular trade was in effect a flywheel: an Atlantic turbine around which new trades developed like eddies.

**Figure 13:**
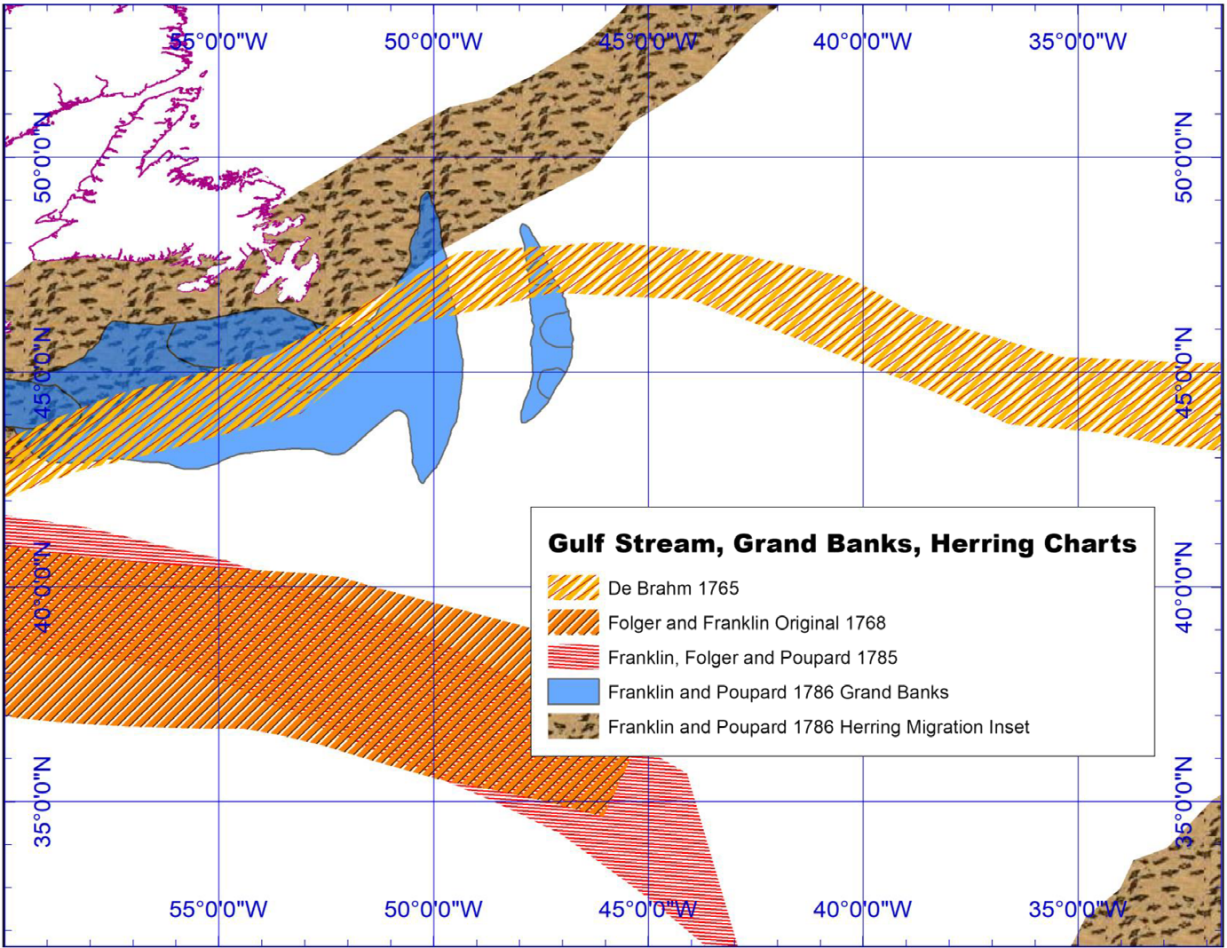
Systems, 1765–1800 (Charles Travis, Kevin Lougheed, Francis Ludlow).

In the late 18th century, nautical charts of the Atlantic and Gulf Stream currents first appeared in William Gerard De Brahm's *The Atlantic Pilot* (1772) and Benjamin Franklin's *Sundry Maritime Observation* (1786) (see Figure 13).

### 6.1 Charting the Atlantic turbine

The Spanish had encountered the current during their early forays into the West Indies. However, the full extent of the Gulf Stream, as an Atlantic gyre, reaching past Newfoundland to the coast of Ireland before doubling back to the Caribbean was not understood. Early chart‐makers who first plotted its course include: British sea captain and colonial tobacco farmer Walter Hoxton; American diplomat, statesman, and scientist Benjamin Franklin; his cousin Timothy Folger, a Nantucket Whaling captain; and colonial Surveyor‐General of South Carolina and Georgia, William Gerard De Brahm (Brown, 1938; Cohn, 2000; De Vorsey, 1976; Peterson et al., 1996).

In 1735, Hoxton drafted a nautical chart of Chesapeake Bay, drawing on latitude sighting and dead reckoning fixes of his tobacco ships’ north–south sets across 23 voyages. He did not draw a “northeast current” on his chart, but noted it possessed a mean speed of 1.3 knots, commensurate with today's measures (Peterson et al., 1996; Richardson, 1982). In 1748, the stream's name was ascribed to the “gulfweed” floating on its current by the Swedish naturalist Pehr Kalm. The earliest cartographical depiction of the Gulf Stream was made by De Brahm in 1765. The observation of its warm, southern current, meandering from the Dry Tortugas around the tip of Florida past present‐day Miami Beach and Key Biscayne by De Brahm is poetic and imagistic:

The Edge of the Florida, Vulgo: Gulf Stream distinguished here by a celadon green, whilst the Stream itself is dark blue & the Waters on the Soundings to the Northward as far as the Rocks are milky white. (De Vorsey, 1976, p. 112)

**Figure 14:**
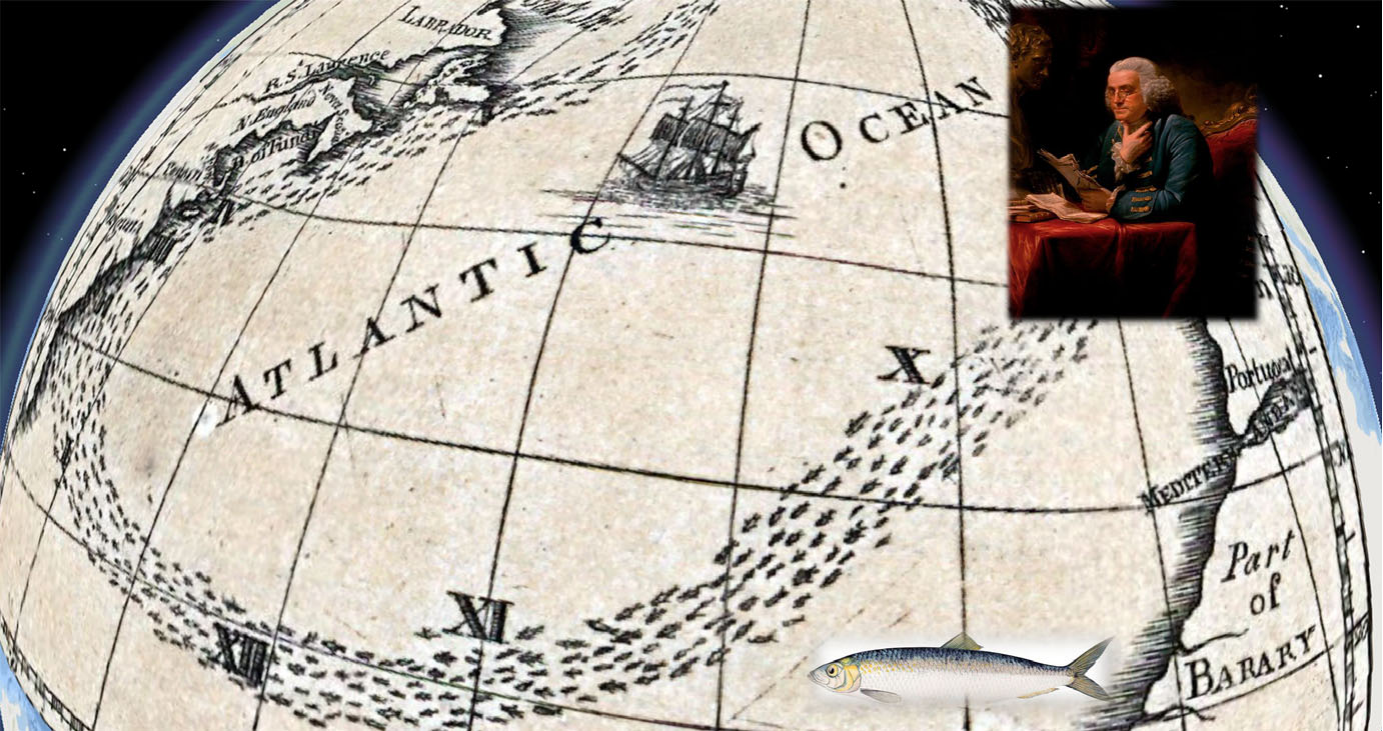
This Herring Migration Chart, was an accidental supplement to Benjamin Franklin and James Poupard’s ‘Gulph Stream Map,’ as the printer was trying to reduce costs. The chart relates to a separate paper on herring migration, however the geo referenced chart corresponds cartographically with the Grand Bank symbolism in the 1786 map published in Franklin’s scientific letter Sundry Maritime Observation. Insets: Portrait of Benjamin Franklin, 1767, Artist David Martin (1737‐1797) and Herring, Sources: Wikicommons. (Charles Travis, Francis Ludlow, Kevin Lougheed).

Franklin's 1769 chart of the Gulf Stream, published for mail packet captains by Mount and Page in London, originated from his position as Deputy Post‐Master General for the American colonies. In “1769 or 1770” he received a query from the Boston Board of Customs, and the Lords of the Treasury in London. Officials were puzzled as to why it took a fortnight longer for mail packets from Falmouth (on the south‐west tip of Cornwall) to sail to New York city than it did for London merchant ships to sail to Rhode Island – especially since the differences in sailing distances favoured the mail packets by a day. Franklin consulted his cousin, Timothy Folger, a Nantucket whaling captain, who replied by drawing a chart, that the packets were sailing into a strong eastward current (Cohn, 2000). Known to New England fishing crews, who sighted whales at its edge, Franklin recorded in *Sundry Maritime Observation* that Folger advised him that “the Rhode Island captains were acquainted with the gulf stream, which those of the English packets were not … but they were too wise to be counselled by simple American fishermen” (Franklin, 1786, p. 314). As envoy of the Continental Congress, Franklin used a thermometer to measure the stream's temperature during voyages to England and France in 1775, 1776, and 1785, observing: “besides the gulph [gulf] weed with which it is interspersed, I find that it is always warmer than the sea on each side of it, and that it does not sparkle in the night” (1786, p. 314) (see Figure 14).

The 1786 version of Franklin and James Poupard's “Gulph Stream” chart, published in *Transactions of the American Philosophical Society* (1786), contains a herring migration inset map, often mistaken for the Gulf Stream though its current intersects and parallels herring spawning and migration flows in the Atlantic (Figure 14). Franklin's Gulf Stream charts can be contextualised by a New England–Newfoundland fishery assemblage preceding and accompanying the American Revolution. From 1768 to 1772, fish represented 35% of New England's total export revenue; by 1775, an estimated 10,000 New Englanders, or 8% of the adult male working population, laboured in the fishing industry (Magra, 2006). With revolutionary tensions coming to a boil in 1775, Britain, aware of economic power of its Grand Banks resource, closed the New England cod‐fishing industry. In 1778 with war between England and the American Colonies underway, Franklin took measures to provide the French navy, allied with the American rebels, his Gulf Stream chart (Philbrick, 2019). Because of the war, New England merchants converted trade routes into military supply lines and transformed fishery tools into naval *matériel*. Fishing vessels became warships with crews manning the first American navy, coast guard, and privateer fleets (Magra, 2006, pp. iv–v).

## 7 CONCLUSION

Because the sea was so thick with cod, brought out by the singing, hundreds of thousands of them, she could walk on them, right across their backs, out and out toward the song …

Emma Hooper, *Our Homesick Songs* (2019)

In 1719, the Jesuit priest Pierre Xavier de Charlevoix, considered the first historian of New France, claimed that the Grand Banks was “properly a mountain, hid under water,” and noted its cod population “seems to equal that of the grains of sand which cover this bank” (Roberts, 2007, p. xxvii). Such hyperbole describing the anchor of the Fish Revolution may be warranted. A preliminary HumGIS model of early modern north‐west Atlantic nautical cartography suggests perceptions of a massive fishery that spanned from Cape Cod to the mouth of the St Lawrence Seaway. More developed HumGIS *distant reading* models would integrate cartographic, archival, and literary texts with historical and archaeological surveys of fishing ports and phytoplankton hindcasting models, not only of cod but also other species such as herring, swordfish, haddock, capelin, and shellfish. Given the speculated extent of this massive fishery, it can be conjectured that the “Grand Banks” was excised and “invented” due to Newfoundland's role as an English oceanic plantation.

HumGIS *close readings* of epistolary “charts” provided by the *Day* and *Pasqualigo Letters* parsed the only cartographic records of Cabot's iconic 1497 voyage. The accidental discovery of a fishery of biblical proportions catalysed the expansion of English sea power and ventures contextualising a north‐west Atlantic reading of *The Tempest.* In turn, Prospero's mysterious island serves as a metaphor for the transformation in perceptions from the mysticism of late medieval cosmology to the empirical nature of early modern scientific thought. The staging of *The Tempest* on the banks of the Thames, a world port, embodied and presaged the visual and literary manifestations of a Western cartographic gaze that began to focus on oceanic and planetary scales during the scientific and republican revolutions of the 18th century. Gulf Stream surveys of oceanic dynamics in De Brahm's *The Atlantic Pilot* (1772) and Franklin's *Sundry Maritime Observation* (1786) serve as examples of the “Brave New World” gaze of period nautical cartography.

**Figure 15:**
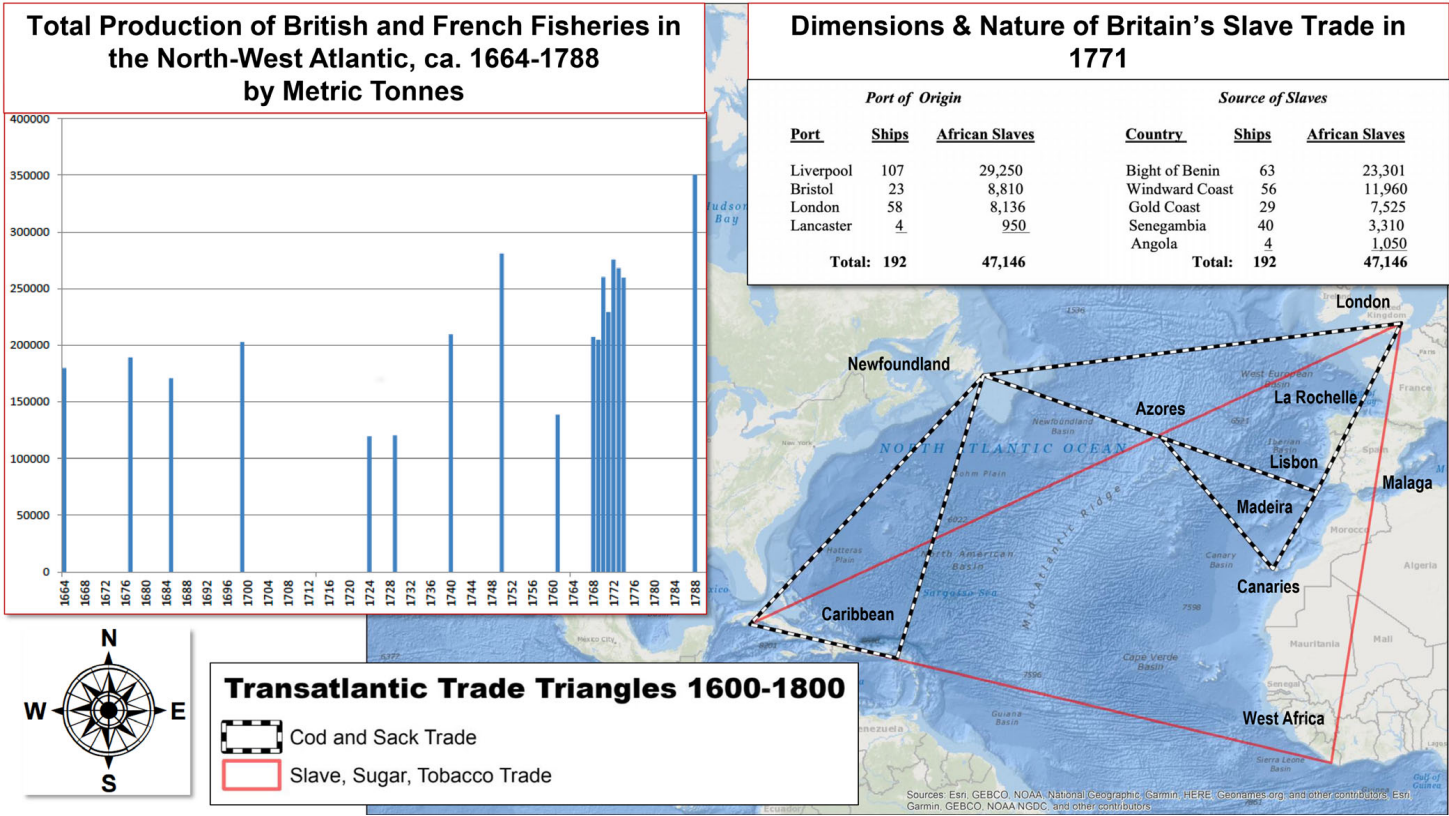
The transatlantic trade triangles ca 1600–1800 (sourced and adapted from Edwards, 1793; Pope, 1996, 2012; Sheridan, 1972; Starkey & Haines, 2017; Turgeon, 2009; Charles Travis).

In conclusion, two main questions have been raised by deep charting the “invention” of the Grand Banks. First, although oceans cover 70% of the earth's surface, they remain largely *aquae incognitae* to human geographers. The study of maritime environments, histories, and cultures would benefit from transdisciplinary work involving marine biologists, oceanographers, historians, geographers, geologists, and scholars in the digital and environmental humanities. Second, more emphasis needs to be placed on the role of the early modern economic engine of the Newfoundland and the Grand Banks “oceanic plantation” in contrast to the iconic, but economically anaemic and failed, colonial settlements at Jamestown and Roanoke. The lucrative cod trade infused the financial capital of London, intersecting with other transatlantic import/export and investment schemes (see Figure 15).

The role of the Cod and Sack Triangle has been elided in transatlantic historiography because of a just and warranted emphasis on the effects of the Slave, Sugar, and Tobacco Triangle (Figure 15). This paper suggests that a deeper integration of the two Atlantic triangle trades could facilitate deeper understandings of relations between massive resource extractions, political‐economic development, and social and environmental justice issues tied to the birth of global modernity. Finally, this study draws upon practices in historical geography, GIScience, and textual analysis to deep chart the “invention” of Grand Banks as a confluence of cartography, commerce, and culture. Further explorations of other regional *aquae incognitae* may help geographers consider historical scales of *ecotonic* resource extractions, and their impacts on 21st century global environmental change.

## Supporting information


**Appendix S1**. 203 Grand Banks Charts 1504–1889. 83 Geo‐rectified charts highlighted in yellow.

## Data Availability

All data can be accessed at https://oceanspast.org/videos/norfish/index.html

## ENDNOTES

1 The *Day and Pasqualigo Letters* (1497–1498), *The Tempest* (1611), and De Brahms (1772) and Franklin's (1786) Gulf Stream surveys were chosen because they represent distinct types of textuality that distinguish the three Braudelian historical cycles composing the *longue durée* during which cultural and environmental factors led to the invention of the Grand Bank. Other commensurate texts include the *Decades of Peter Martyr* (1515), which included an account of Cabot's 1497 voyage. Richard Hakluyt’ *Divers voyages touching the discovery of America* (1582) preceded, but celebrated in anticipation of Humphrey Gilbert's declaration of Newfoundland as an English colonial possession in 1583. In the 17th century, with the establishment of the “oceanic plantation,” Richard Whitbourne's *Discourse of Newfoundland* (1620), William Vaughn's *Golden Fleece* (1626) and Robert Hayman's book of verse *Quodlibets* (1628) were published to promote the island's colonisation. From the 19th century onwards, Herman Melville's *Redburn: His First Voyage* (1849), Rudyard Kipling's *Captains Courageous* (1897), Tom Clancy's *The Hunt for Red October* (1984), Sebastian Junger's *The Perfect Storm* (1997), and Emma Hooper's *Our Homesick Songs* (2018) represent different perspectives on the geo‐political, economic, and *ecotonic* dimensions of the Grand Banks, Newfoundland island, and the wider Northwest Atlantic.

2 As Peter Pope (2012, p. 95) observes, “‘Sack’ derives from *vino de sacca*, or wine set aside for export, rather than from *vino secco*, or dry wine.”

3 This study's survey of Grand Banks fishery charts (of which there are a prolific amount) focus on those published between 1500 and 1900, and betray a dominant Anglo‐Franco colonial perspective. Collections surveyed include The British Library, The Library of Congress, Memorial University of Newfoundland, John Carter Brown Library, Norman B. Leventhal Map center at the Boston Public Library, the Huntington Library, and the Gallica and David Rumsey Collections. We selected charts with unambiguous fishery symbolism. We were interested in the morphology and the range indicated by fishery symbolism as it evolved between the 14th and 19th centuries. We were also interested in the location of chart publications. Major nautical cartography collections in Italy, Germany, The Netherlands, Scandinavia, Portugal, and Spain were not explored. Though we found indigenous Beothuk mappings of terrestrial Newfoundland, we could find no indigenous fishery representations. We also found little production in terms of colonial nautical cartographers, and except for DeBrahms and Franklin, only a few charts were produced from an Americanist perspective prior to and following the period of the colonial revolution in 1776.

4 This method preserves the collinearity of point relations and the ratios of distances along a line.

5 Spearman's rank coefficient was preferred to Pearson's coefficient for the present data because of outlying values likely to otherwise bias the result.

6
Pope (n.d.).
