# Supplementary material for: Inventing the Grand Banks: A deep chart: Humanities GIS, Cartesian, and literary perceptions of the north‐west Atlantic fishery ca 1500–1800
Source: Geo. 2020 Mar 30;7(1):e00085. doi: 10.1002/geo2.85 (PMC9286359; doi:10.1002/geo2.85)
Supplement: Supplementary file 1 — Appendix S1. 203 Grand Banks Charts 1504–1889. 83 Geo‐rectified charts highlighted in yellow. [file GEO2-7-0-s001.docx]

**Appendix 1.**

| **Deep Chart Table 1: 203 Grand Banks Charts 1504-1889. 83 Geo-rectified charts highlighted in yellow** | | |
| --- | --- | --- |
| **Year** | **Cartographer** | *Official Title* and **City of Publication** |
| **1504** | Pedro Reinel | Portulan (*Atlantik*) (*Kunstmann I*) **Lisbon** |
| **1508** | Johannes Ruysch | *Universalior Cogniti Orbis Tabula. Ex recentibus confecta observationibus* [A Map of the Known World, according to the newest discoveries]., **Rome** |
| **1516** | Vesconte Maggiolo | [Portolan atlas], **Naples** |
| **1529** | Girolamo de Verrazano | [Mapamundi], **Dieppe** |
| **1529** | Diego Ribiero | *Carta universal en que se contierne todo lo que del mundo se ha descubierto fasta agora hizola Diego Ribero cosmographo de Su magestad. Año de 1529 è Sevilla. La qual Se devide en dos partes conforme a la capitulcio que hizieron los catholicos Reyes de españa, y El Rey don Juan de portugal e la Villa de tordessilas: Año de 1494* [General chart containing the whole of the world that has hitherto been discovered; complied by Diego Ribero, cosmographer to His Majesty, which is divided into two parts according to the agreement made by the Catholic Majesties of Spain and King John of Portugal at Tordessilas, A.D. 1494]. **Seville** |
| **1531** | Vesconte Maggiolo | *The Vesconte Maggiolo Planisphere of 1531,* **Genoa** |
| **1541** | Nicholas Desliens | *Desliens World Map, with North-up Orientation, Showing a Portion of North America, ca. 1541,* **Dieppe** |
| **1542** | Jean Rotz | *Boke of Idrography* (The 'Rotz Atlas'), **Dieppe** |
| **1542** | Battista Agnese | *Nautical Atlas*, **Genoa** |
| **1543** | Guillaume Brouscon | [Nautical almanac] Folio binding, ***Le Conquet*** |
| **1544** | Sebastian Cabot | *World map of A.D. 1544* (the Sebastian-Cabot map) and 1882 facsimile [*Océan Atlantique nord. Reproduction grandeur de l'original d'une partie de la mappemonde de 1544 / par Sébastien Cab*or] *Fac-simile par J. Pilinski, d'après l'exemplaire unique conservé au Département des cartes de la Bibliothèque nationale de Paris,* **Paris** |
| **1546** | Pierre Desceliers | *Mappemonde,* **Calais** |
| **1550** | Diego Gutierrez | *Carte de l'Océan Atlantique, Diego gutierrez cosmographo de Su magd me fizo en sevilla Ano de 1550,* **Seville** |
| **1555** | Guillaume Le Testu | *Cosmographie Universelle,* **Dieppe** |
| **1556** | Giocomo Gastaldi | *La Nvova Francia,* **Venice** |
| **1560** | Bartolomeu Velho | [Portolan atlas] f4, **Lisbon** |
| **1562** | Diego Gutierrez | *Americae Sive Qvartae Orbis Partis Nova Et Exactissima Descriptio,* **Antwerp** |
| **1564** | Antoine Du Pinet *et. al.* | *Carte cosmographique,* **Lyon** |
| **1570** | Abraham Ortelius | *Theatrum orbis terrarum* (Image 18), **Antwerp** |
| **1570** | Abraham Ortelius | *Septentrionalium Regionum Descrip. Theatrum orbis terrarum* (Image 106) |
| **1570** | Fernão Vaz Dourado | [Portolan atlas] f4, **Goa** |
| **1572** | Jaime Holives | *Map of the Atlantic Ocean,* **Barcelona** |
| **1573** | Abraham Ortelius | *Septentrionalium Regionum Descrip. “Theatrum orbis terrarium”*, **Antwerp** |
| **1573** | Domingos Teixeira | [Planisphère], **Lisbon** |
| **1575** | André Thevet | *Le nouveau monde descouvert et illustre de nostre temps,* **Paris** |
| **1576** | Humphrey Gilbert | *A General Map, Made Onelye for the Particuler Declaration of this Discovery,* **London** |
| **1578** | George Best | [Untitled Oval Map of the World] **London** |
| **1578** | George Best & James Beare | [Untitled map of Arctic and Northwest Passage] **London** |
| **1578** | Joan Martines | [Portolan atlas] f6, **Messina** |
| **1580** | John Dee | *A spherical projection of a part of the northern hemisphere, including all America north of the line, and the western parts of Europe and Africa, by John Dee, 1580*, very neatly drawn, on vellum. **London** |
| **1582** | Michael Lok | *Illustri viro, domino Philippo Sidnæo Michael Lok civis Londinensis hanc chartam dedicabat: 1582,* **London** |
| **1582** | Joan Martines | *Atlas nautique du monde ext em de sept cartes manuscrites sur vélin,* **Messina** |
| **1583** | John Dee | *The Gilbert Map c.1583* (Reproduction of the ms map of North America and the Arctic Regions by Sir H. Gilbert c. 1583) **London** |
| **1587** | Richard Hakluyt & Edward Wright | [Map of the World], **Paris** |
| **1588** | Bartolomeo Lasso | *Portulan des mers d’Europe et du nord de l’océan Atlantique,* **Lisbon** |
| **1589** | Baptista Boazio | [Map and views illustrating Sir Francis Drake’s West Indian voyage, 1585-6] **London** |
| **1590** | Pedro de Lemos & Sebastião Lopes | *Planisphère,* **Lisbon** |
| **1592** | Petrus Plancius | *Nova Francia, alio ext em dicta Terra nova : anno 1504 à Britonibus primum detecta,* **Amsterdam** |
| **1593** | Cornelis de Jode | *Americae pars borealis, Florida, Baccalaos, Canada, Corterealis,* **Antwerp** |
| **1594** | Cornelis Claesz | *Nova Francia, alio ext em dicta Terra nova : anno 1504 à Britonibus primum detecta*  **Amsterdam** |
| **1594** | Petrus Plancius | *Orbis ext ems typus de integro multis in locis emendates,*  **Amsterdam** |
| **1598** | Barrent Langenes | *Terra Nova,* **Middelburg** |
| **1599** | Jan Dirckx | *Carte de l’Océan Atlantique nord,*  **Amsterdam** |
| **1600** | Edward Wright | *Map of the world A.D. 1600,* **London** |
| **1602** | Petrus Bertius | *Terra Nova,* **Amsterdam** |
| **1606** | Willem Janszoon Blaeu | *Nova totius ext ems orbis geographica ac hydrographica tabula,*  **Amsterdam** |
| **1608** | C. Stokes | *The Virginia Company Chart,* **London** |
| **1609** | Marcvs Lescarbot | *Figvre de la terre nevve, grande riviere de Canada, et côtes de l’ocean en la Novvelle France,* **Paris** |
| **1611** | Jodocus Hondius | *Novissima ac exactissima totius orbis ext ems ext emse magna,* **Amsterdam** |
| **1612** | Samuel de Champlain | *Carte geographique de la Nouvelle Franse,* **Paris** |
| **1617** | Unknown | *Terre Neuve* (Division 2 du portefeuille 128 du Service hydrographique de la marine consacrée aux cartes générales de l’île de Terre – Neuve), **Dieppe** |
| **1618** | La Bocage-Boissaie | *Partie de Terre – Neuve,* **Le Havre** |
| **1624** | Paul Ollivier | *Carte de l’Océan Atlantique Nord / Faite a Grâce par Paul Ollivier année 1624,* **Le Havre** |
| **1625** | John Mason | *Newfound Land,* **London** |
| **1625** | William Alexander | *New France, New Englande, New Scotlande, and New Foundlande,* **London** |
| **1625** | Samuel Purchas | *Map showing New France, New England, New Scotlande and New Found Land,* **London** |
| **1630** | Anonymous | Portolan Atlas, **Macao** |
| **1643** | Jean Boisseau | *Description de la Nouvelle France : ou sont remarquées les diverses habitations des François, despuis la première descouverte jusques a present, receuillie et dressée sur diverses relations modernes,* **Paris** |
| **1647** | Robert Dudley | *Carta particolare della terra nuoua con la Gran Baia et il Fiume Grande della Canida,* **Florence** |
| **1650** | Jan Jansson | *Mar del Nort,* **Amsterdam** |
| **1650** | Unknown | *Carte basque de l’isle de Terre Neuve, de la Cadie et Canada,* **Dieppe** |
| **1650** | Unknown | *Roche découverte sur le banc de Terre Neuve,* **Dieppe** |
| **1650** | Unknown | *Plan basque des côtes de Terre – Neuve , des côtes de l’Acadie et des bancs environnants,* **Dieppe** |
| **1650** | Unknown | *Carte du grand banc et golfe St Laurent,* **Dieppe** |
| **1656** | Nicolas Sanson | *Le Canada, ou Nouvelle France, &c. ce qui est le plus advance vers le septentrion est ext de diverses relations des Anglois, Danois, &c. vers le ext les ext de Virginie, Nouv[e]lle Suede, Nouveau Pays Bas, et Nouvelle Angleterre sont tirées de celles des Anglois, Hollandois, &c. La Grande Riviere de Canada ou de St Laurens, et tous les environs sont suivant les relations des Francois.,* **Paris** |
| **1660** | Pieter Goos | *West-Indische paskaert waer in de graden der breedde over weder zijden vande middellijn wassende so vergrooten dat die geproportioneert sijn tegen hunne nevenstaende graden der lengde,* **Amsterdam** |
| **1660** | Frederick de Wit | *Nova totivs Americæ description,* **Amsterdam** |
| **1660** | Hendrick Doncker (pub.) | *Pascaerte van Brazil en Nieu Nederlandt, van Corvo en Flores tot de Barbados,*  **Amsterdam** |
| **1660** | Nicolosi Giovanni Battista | *Mexicvm,* **Rome** |
| **1662** | Joan Blaeu | *Extrema Americae versus Boream, ubi Terra Nova Nova Francia, adjacentiag,*  **Amsterdam** |
| **1664** | Pierre Duval | *Le Canada faict par le Sr. de Champlain, ou sont la Nouvelle France, la Nouvelle Angleterre, la Nouvelle Holande, la Nouvelle Svede, la Virginie &c. avec les nations voisines et autres terres nouvellement decouvertes / suivant les memoires de P. Du Val, geographe du Roy, a Paris en l’Isle du Palais,* **Paris** |
| **1665** | Hendrick Doncker (pub.) | *Pascaerte van Terra Nova, Nova Francia, Nieuw Engeland en de groote revier van Canada,* **Amsterdam** |
| **1671** | John Seller | *A chart of the northerne sea, from England westerly as farr as New Found Land and northerly as farr as Island Groenland and Fretum Davis,* **London** |
| **1673** | John Thornton | *A new mapp of the north part of America : from Hudson Straights ext ems call’d the Norwest Passage, including Newfoundland, New Scotland, New England, Virginia, Maryland & Carolena,* **London** |
| **1674** | Moy Denis de Rotis | *Carte de l’Océan Atlantique nord,* **St-Jean de Luz** |
| **1675** | John Seller | *A chart of the coast of America from New found Land to Cape Cod,* **London** |
| **1675** | John Thornton | *A Chart of the Coast of America from New found Land to Cape Cod,* **London** |
| **1676** | Courcelle, de | *Carte des côtes de Terre-Neuve,* **Dieppe** |
| **1677** | William Hacke | *Map of New France, Gulf of St. Laurence, New Scotland, Accadia and New found land,* **London** |
| **1678** | Georges Boissaye du Bocage | *Partie de Terre Neuve, Grand Banc,* **Le Havre** |
| **1683** | Allain, Manesson-Mallet | *Canada ou Nouvelle France,* **Paris** |
| **1683** | Allain, Manesson-Mallet | *Isle de Terre Neuve,* **Paris** |
| **1685** | Johannes van Keulen | *Pascaarte vande Noorder Zee custen van America : vande West-hoeck van Ysland ext e Straet Davis en Hudson, tot aen Terra Neuf,* **Amsterdam** |
| **1689** | John Thorton | *The English Pilot Charts 4^th^ book ‘A New Chart of the Sea Coast of Newfoundland, new Scotland, new England, new Jerfey, Virginia, Maryland, Pennfilvaniam and part of Carolina’,* **London** |
| **1689** | Vincenzo Coronelli | *Partie orientale du Canada ou de la Nouvelle France,* **Paris** |
| **1690** | John Seller | *A chart of the coast of America from Newfound Land to Cape Cod,* **London** |
| **1690** | John Seller | *New Found Land,* **London** |
| **1692** | Vincenzo Coronelli | *Canada orientale nell’ America settentrionale,* **Venice** |
| **1693** | Pierre Mortier | *Le Canada ou partie de la Nouvelle France,* **Amsterdam** |
| **1693** | Augustine Fitzhugh | *A chart of the coasts of Newfoundland, with the fishing districts marked; “by Augustine Fitzhughe, living next doare to the Shipp in Virgine Street, anno 1693”,* **London** |
| **1696** | Nicolas Sanson | *Partie orientale de l’Amerique angloise,* **Amsterdam** |
| **1696** | Nicolas Sanson | *Le Canada, ou, Partie de la Nouvelle France : contenant la Terre de Labrador la Nouvelle France, les isles de Terre Neuve, de Nostre Dame, &c.,* **Amsterdam** |
| **1696** | Le Cordier, Roussel, Hubault | *Carte de la baye de Canadas, de la riviere de Kebec, du banc de terre neuve,* **Le Havre** |
| **1697** | Vincenzo Coronelli | *Canada orientale nell’ America Settentrionale,* **Venice** |
| **1697** | Blaise Vion | *Plan de l’entrée du Grand Burin, Petit Burin et de la baye de la Saumonnière,* **Dieppe** |
| **1698** | Jacques Chaviteau | *Carte de l’isle de Terre-Neuve,* **Dieppe** |
| **1698** | John Thornton | *A chart of ye iland of New Found Land,* **London** |
| **1698** | John Thornton | *A new chart of the trading part of New Found Land,* **London** |
| **1698** | John Thornton | *A generall chart of the West Indies,* **London** |
| **1698** | John Thornton | *A chart of ye north part of America,* **London** |
| **1699** | John Thornton | *A new and correct chart of the north part of America,* **London** |
| **1700** | Guillaume de l’Isle | *L’Amerique Septentrionale Dressee sur les Observations de Mes. De L’Academie Royale des Sciences… America Septentrionalis in Suas Praecipuas Partes Divisa, ad usum Serenissumu Burgundiae Ducis,* **Paris** |
| **1700** | Pierre Mortier | *Partie Orientale de l’Amérique Angloise,* **Amsterdam** |
| **1700** | Thomas Bonaventure Godalles | *Plan Geometral de l’isle de terre-neuve &c du grand Banc, & Banc Jacquet, que des autres isles & Bancs, qui luy sont adjacens, a Bise proche la coste de la nouvelle France en l’Amerique Septentrionale,* **St. Malo** |
| **1700** | Unknown | *Sud de Terre-Neuve et île Royale,* **Dieppe** |
| **1702** | Mount and Page | *A chart of the banks and harbours of Newfoundland,* **London** |
| **1702** | Nicolas de Fer &  Herman Van Loon | *Le Canada ou Nouvelle France, la Floride, la Virginie, Pensilvanie, Caroline, Nouvelle Angleterre et Nouvelle Yorck, l’isle de Terre Neuve , la Louisiane et le cours de la rivière de Misisipi,* **London** |
| **1705** | Nicolas de Fer | *L’Amerique, divisee selon l’etendue de ses Principales Parties, et dont les Points Principaux sont placez sur les Observations de Messieurs de L’Academie Royale des Sciences,* **Paris** |
| **1707** | Pieter van der Aa | *Zee-togten door Thomas Candys na de West Indien : en van daar rondom den gantzen aardkloot gedaan,* **Leyden** |
| **1708** | Guillaume de l’Isle | *L’Amerique Septentrionale Dressee sur les Observations de Mes. De L’Academie Royale des Sciences,* **Amsterdam** |
| **1708** | Jean Baptiste Nolin | *Le globe terrestre représenté en deux plans-hémisphères,* **Paris** |
| **1708** | Jean Baptiste Nolin | *Le globe terrestre représenté en deux plans-hémisphères : dressé sur la projection de Mr. de la Hyre de l’Académie Royale des Sciences, et sur plusieurs routiers et mémoires des plus habiles pilotes et savans voyageurs le tout rectifié et calculé selon les dernières observations, et dédié à Mgr. l’Abbé Bignon, conseiller d’état ordinaire,* **Paris** |
| **1710** | Thomas Bonaventure Godalles | *Succinte et Tres veritable, & authentique relation de la decouverte, .de l’isle de terre-neuve, le grand Banc, & Banc-Jacquet,* **St. Malo** |
| **1711** | P.Mortier (eng.) | *Extrema Americae versus Boream,* **Amsterdam** |
| **1712** | Herman Moll | *A map of New France containing Canada, Louisiana &c. in N[or]th. America according to the patent granted by the King of France to Monsieur Crozat, dated the 14^th^ of Sep. 1712 n.s. and registered in the Parliament of Paris the 24^th^ of the same month by H. Moll, geographer,* **London** |
| **1713** | Anon or Unknown | *Plan géométral de l’isle de Terre-Neuve et autres isles et bancs,* **Dieppe** |
| **1718** | Nicolas Visscher | *Carte nouvelle contenant la partie d’Amerique la plus septentrionale : ou sont exactement dêcrites les provinces suivantes comme le Canada ou Nouvelle France, la Nouvelle Ecosse, la Nouvelle Angleterre, les Nouveau Païs Bas, la Pensylvanie, la Virginie, la Caroline, et l’Ile de Terre Neuve, avec les profondeuis de long des côtes et sur les bancs,* **Amsterdam** |
| **1720** | Henry Moll | *Map of North America,* **London** |
| **1720** | Nicolaes Visscher | *Nova tabula geographica complectens Borealiorem Americæ partem in qua exacte delineatæ sunt Canada sive Nova Francia, Nova Scotia, Nova Anglia, Novum Belgium, Pensylvania, Virginia, Carolina, et Terra Nova, cum omnibus littorum, pulvinorumque profunditatibus,* **Amsterdam** |
| **1721** | Michel Bremond | *Plan de l’ille Royalle, de l’ille de Terre Neuve et du golf de Saint Laurent,* **Dieppe** |
| **1725** | M. Du. Coulombier (eng.) | *Carte du grand banc et batures des ext et isle de Terre-Neuve,* **Dieppe** |
| **1728** | Gerard van Keulen | *Nouvelle Carte Marine du Grand Banq de Terra Neuff a Grand Point,* **Amsterdam** |
| **1730** | Homann Erben | *Dominia Anglorum in America Septentrionali Specialibus Mappis Londini primum a Mollio edita, nunc recusa ab Homannianis Hered,* **Nuremberg** |
| **1732** | Herman Moll | *New Found Land, St. Laurence Bay, the fishing banks, Acadia, and part of New Scotland,* **London** |
| **1733** | Henry Popple | *A map of the British Empire in America with the French and Spanish settlements adjacent thereto [sheet 8],* **London** |
| **1733** | Henry Popple | (*Composite Map of) A Map of the British Empire in America with the French and Spanish Settlements adjacent thereto. By Henry Popple. C. Lempriere inv. & del. B Baron Sculp. To the Queen’s Most Excellent Majesty This Map is most humbly Inscribed by Your Majesty’s most Dutiful, most Obedient, and most Humble Servant Henry Popple. London Engrav’d by Willm. Henry Toms & R.W. Seale, 1733.,* **London** |
| **1733** | Jacques Chaviteau | *Carte particulière de la rivière de Québec,* **Dieppe** |
| **1736** | Liébaut | *Carte du Grand Banc, Terre Neuve et golfe de St Laurent,* **Dieppe** |
| **1736** | Philippe Buache | *Carte des côtes méridionales de l’isle de Tere Neuve comprenant les Isles Royales et de Sable avec la partie du Grand Banc, où se fait la pêche de la morue,* **Paris** |
| **1737** | Anon or Unknown | *Carte pour faire voir la route du …Jason en 1737,* **Dieppe** |
| **1737** | Homann Erben | *Dominia Anglorum in America Septentrionali : specialibus mappis Londini primum a Mollio edita, nunc recusa ab Homannianis Hered,* **Nuremberg** |
| **1738** | Gabriel Pellegrin | *Carte du golffe de St Laurent et coste du sud de l’isle de Terre Neuve,* **Dieppe** |
| **1741** | Philippe Buache | *Carte des côtes méridionales de l’isle de Terre Neuve comprenant les Isles Royale et de Sable avec la partie du Grand Banc, où se fait la pêche de la morue / dressée par Philippe Buache et ext em avec le plan de la carte de Mr. Popple en l’an 1733,* **Paris** |
| **1743** | Jacques Nicolas Bellin | *Carte de L’Amerique Septentrionale,* **Paris** |
| **1744** | Jacques Nicolas Bellin | *Carte de l’Isle de Terre-Neuve,* **Paris** |
| **1745** | Jacques Nicolas Bellin | *Partie orientale de la Nouvelle France ou du Canada,* **Paris** |
| **1751** | M. de Chabert | *Carte Reduite Des Costes,* **Paris** |
| **1752** | Emanuel Bowen | *A new & accurate map of the islands of Newfoundland, Cape Breton, St. John and Anticosta; together with the neighbouring countries of Nova Scotia, Canada, &c.,* **London** |
| **1753** | Joseph Bernard de Chabert | *Carte Reduite,* **Paris** |
| **1753** | Seligny | *Plan de l’atterage de Louisbourg et partie de Terre Neuve et du grand banc,* **Dieppe** |
| **1754** | M. Rouelle | *Carte réduite du golphe de St Laurent,* **Dieppe** |
| **1755** | Jean Palairet | *Carte de l’Amérique septentrionale, 1754,* **London** |
| **1755** | John Mitchell | *A map of the British and French dominions in North America, with the roads, distances, limits, and extent of the settlements, humbly inscribed to the Right Honourable the Earl of Halifax, and the other Right Honourable the Lords Commissioners for Trade & Plantations,* **London** |
| **1755** | London Magazine | *A map of the British & French Plantations in North America,* **London** |
| **1755** | Robert de Vaugondy | *Nouvelle France ou le Canada,* **Paris** |
| **1755** | Thomas Jefferys | *Partie orientale du Canada,* **Paris** |
| **1756** | Inglezes | *Mapa de un parte de la American Septentriol,* **Paris** |
| **1756** | Jean Baptiste Bourguignon d’Anville | *America Septentrionalis a Domino d’Anville in Galliis edita nunc in Anglia. Coloniis in interiorem Virginiam deductis nec non Fluvii Ohio  cursu aucta notisque geographicis et historicis ext emse.,* **Nuremberg** |
| **1759** | James Turner | *This map of the province of Nova-Scotia and parts adjacent* |
| **1762** | Guillaume de l’Isle | *America septentrionalis concinnata juxta observations,* **Augsburg** |
| **1762** | Jean Denis Janvier | *L’Amérique septentrionale divisée en ses principaux états,* **Paris** |
| **1763** | Jacques Nicolas Bellin | *Carte de l’isle Saint Pierre,* **Paris** |
| **1763** | James Cook | *A sketch of the island of Newfoundland,* **St. John’s** |
| **1764** | Jacques Nicolas Bellin | *Carte Reduite Du Grand Banc et d’une partie de l’isle de terre neuve,* **Paris** |
| **1766** | Jacques-Nicolas Bellin | *Carte réduite de l’Ocean occidental contenant partie des ext d’Europe et d’Afrique : depuis le 51e dégré de latitude septentrionale jusqu’à l’Equateur et celles de l’Amerique qui leur sont ext em ,* **Paris** |
| **1768** | Benjamin Franklin &  James Folger | *Franklin-Folger chart of the Gulf Stream,* **London** |
| **1768** | Richard Seale | *A new and accurate map of North America, drawn from the famous Mr d’Anville with improvements from the best English maps : also the new divisions according to the late Treaty of Peace,* **London** |
| **1770** | Antonio Zatta | *L’America Divisa Nei Suoi Principali Stati, Di nuova projezione,* **Venice** |
| **1770** | Robert Sayer | *A chart of the South-East part of Newfoundland,* **London** |
| **1771** | Carington Bowles | *A new chart of the vast Atlantic or Western Ocean including the sea coast of Europe, Africa, America, and the West India Islands with the banks, shoals, rocks & course of sailing from one continent to the other, laid down from the latest discoveries & regulated by numerous astronomical observations,* **London** |
| **1771** | Georges Boissaye du Bocage | *Le grand banc de Terre Neuve,* **Le Havre** |
| **1772** | Charles-Pierre Claret de Fleurieu | *Nouvelle carte réduite de l’océan Atlantique ou occidental,* **Eveux** |
| **1772** | J. Derauche (eng.) | *Carte du Grand Banc de Terre Neuve,* **Paris** |
| **1772** | M. Duhamel du Monceau | *Plan figuré des différens Parages ou l’on fait la Pesche de la Morue,* **Paris** |
| **1773** | Unknown | *Côte Sud-Est de Terre-Neuve,* **Dieppe** |
| **1774** | Jacques Nicolas Bellin | *Carte de l’océan occidental et partie de l’Amérique septentrionale dressée pour l’intelligence du journal du voyage que le R.P. de Charlevoix de la Compagnie de Jesus a fait en 1720 au Canada, à la Louisiane & à St Domingue,* **Paris** |
| **1774** | Samuel Dunn | *North America, as divided amongst the European powers,* **London** |
| **1775** | Joseph Bernard Chabert | *A chart of the Banks of Newfoundland,* **London** |
| **1775** | Robert Sayer | *A chart of the gulf of St Laurence.* **London** |
| **1775** | Robert Sayer | *A chart of the banks of New Foundland,* **London** |
| **1776** | James Turner | *This map of the province of Nova-Scotia and parts adjacen.,* **Philadelphia** |
| **1776** | John William De Brahm | *Chart of the Atlantic Ocean,* **London** |
| **1776** | Robert Sayer & John Bennett | *A general map of the northern British colonies in America. Which comprehends the province of Quebec, the government of Newfoundland, Nova-Scotia, New-England and New-York,* **London** |
| **1776** | Samuel Dunn | *A map of the British empire, in North America,* **London** |
| **1776** | Thomas Jeffreys | *A Chart Of The Banks Of Newfoundland,* **London** |
| **1779** | Joseph F. W. Des Barres | *The Coast of Nova Scotia, New England, New York, Jersey, the Gulph and River of St. Lawrence, the islands of Newfoundland, Cape Breton, St. John, Antecosty, Sable, &c, and soundings thereof.,* **London** |
| **1780** | Rigobert, Bonne | *L’Isle de Terre-Neuve, l’Acadie, ou la Nouvelle Ecosse,* **Geneva** |
| **1783** | Gerard Hulst van Keulen | *Chart of the banks of Newfoundland, drawn from a great number of hydrographical surveys, these charts are followed, according to them executed by order of the Lords Commissioners of the Admiralty in England,* **Amsterdam** |
| **1784** | Anon or Unknown | *Carte réduite des bancs et de l’île de Terre-Neuve [avec ext ems. Sur les traités de 1713, 1763, 1783],* **Dieppe** |
| **1784** | James Cook | *Carte réduite des bancs et de l’île de Terre-Neuve,* **Paris** |
| **1784** | James Cook | *Carte réduite de l’île de Terre-Neuve ,* **Paris** |
| **1784** | Thomas Kitchin | *A map of New England and Nova Scotia,* **London** |
| **1785** | Georges-Louis Le Rouge | [*Franklin–Folger map*] *Remarques sur la navigation de terre-neuve à New-York afin d’eviter les courrants et les bas-fonds au sud de Nantuckett et du Banc de George,* **Paris** |
| **1786** | Benjamin Franklin &  James Poupard | *A chart of the Gulf Stream,* **Philadelphia** |
| **1787** | Benjamin Franklin | *Remarks upon the Navigation from Newfoundland to New York In order to avoid the Gulph Stream,* **London** |
| **1793** | Gilles Robert de Vaugondy | *Partie de l’Amérique septent? Qui comprend la Nouvelle France ou le Canada,* **Paris** |
| **1794** | Samuel Dunn | *A General Map of the World, or Terraqueouis Globe with all the New Discoveries and Marginal Delineations, Containing the Most Interesting Particulars in the Solar, Starry and Mundane System.,* **London** |
| **1795** | William Heather | *A New and improved chart of the gulf and river of St. Laurence,* **London** |
| **1795** | William Heather | *Newfoundland and the Great Bank,* **London** |
| **1797** | William Heather | *A new chart of the Atlantic or Western Ocean,* **London** |
| **1802** | William Strickland | *A Chart of the Atlantic or Western Ocean,* **Philadelphia** |
| **1810** | Josef Espinosa y Tello | *Carta general del Oceano Atlantico Septentrional construida con presencia de las publicadas por la direccion Hidrografica de Madrid, y otros conocimientos modernos. Por D[o]n. Jose de Espinosa gefe de esquadra de la Real Armada,* **London** |
| **1817** | John Thomson | *Chart of North Atlantic Ocean with tracks of the shipping to West Indies, North America,* **Edinburgh** |
| **1819** | J. Cary | *A new map of Nova Scotia, Newfoundland,* **London** |
| **1820** | Unknown | *Newfoundland and Nova Scotia,* **Weimar** |
| **1821** | C. [sic] A. Anspach | *Charte der Baenke von Newfoundland,* **Weimar** |
| **1825** | Philippe van der Maelen | *Grand Banc de Terre-Neuve* **Brussels** |
| **1831** | William Hooker | *Chart of the Atlantic Ocean : with an illustration of the character and rout[e] of a storm which occurred on the American coast in August 1830,* **New York** |
| **1833** | Ales (eng.) | Ile et banc de Terre-Neuve avec Iles St. Pierre et Miquelon, **Paris** |
| **1838** | M. Blachford | *A new chart of Newfoundland, and coast of Labrador extending to Sandwich Bay,* **London** |
| **1854** | M.F. Maury | *Depths of the Ocean: Reproduction of Lieut. Maury's Map of North Atlantic, 1854,* **Philadelphia** |
| **1888** | A.C. Roberts | *Limits under fishery treaties, 1818 and 1888,* **Washington, D.C.** |
| **1889** | Julien Thoulet | *Cartes bathymétriques et géologiques des bancs de Terre Neuve,* **Paris** |
